# Nutritional status and prey energy density govern reproductive success in a small cetacean

**DOI:** 10.1038/s41598-021-98629-x

**Published:** 2021-11-02

**Authors:** Lonneke L. IJsseldijk, Sanne Hessing, Amy Mairo, Mariel T. I. ten Doeschate, Jelle Treep, Jan van den Broek, Guido O. Keijl, Ursula Siebert, Hans Heesterbeek, Andrea Gröne, Mardik F. Leopold

**Affiliations:** 1 Division of Pathology, Department of Biomolecular Health Sciences, Faculty of Veterinary Medicine, Utrecht University, Utrecht, The Netherlands; 2 Wageningen Marine Research, Den Helder, The Netherlands; 3 Scottish Marine Animal Stranding Scheme, Inverness, UK; 4 Information and Technology Services, Utrecht University, Utrecht, The Netherlands; 5 Department of Population Health Sciences, Faculty of Veterinary Medicine, Utrecht University, Utrecht, The Netherlands; 6 Institute for Terrestrial and Aquatic Wildlife Research, University of Veterinary Medicine Hannover, Büsum, Germany

**Keywords:** Zoology, Ecology, Environmental sciences, Ocean sciences, Marine biology, Conservation biology, Ecology, Population dynamics

## Abstract

A variety of mammals suppress reproduction when they experience poor physical condition or environmental harshness. In many marine mammal species, reproductive impairment has been correlated to polychlorinated biphenyls (PCBs), the most frequently measured chemical pollutants, while the relative importance of other factors remains understudied. We investigate whether reproductively active females abandon investment in their foetus when conditions are poor, exemplified using an extensively studied cetacean species; the harbour porpoise (*Phocoena phocoena*). Data on disease, fat and muscle mass and diet obtained from necropsies in The Netherlands were used as proxies of health and nutritional status and related to pregnancy and foetal growth. This was combined with published life history parameters for 16 other areas to correlate to parameters reflecting environmental condition: mean energy density of prey constituting diets (MEDD), cumulative human impact and PCB contamination. Maternal nutritional status had significant effects on foetal size and females in poor health had lower probabilities of being pregnant and generally did not sustain pregnancy throughout gestation. Pregnancy rates across the Northern Hemisphere were best explained by MEDD. We demonstrate the importance of having undisturbed access to prey with high energy densities in determining reproductive success and ultimately population size for small cetaceans.

## Introduction

The evolutionary causes and consequences of adaptions in life history strategies shape reproductive success and, consequently, long-term survival^1–3^. Most large mammals, ranging from whales and elephants to humans, are typical K-strategists that maximise lifetime reproductive output by having a low reproductive rate but high survivorship^2–4^. Despite this, life history traits may vary spatiotemporally due to variance in social or cultural, ecological, and environmental factors^3^, as demonstrated in a variety of mammals known to suppress reproduction in response to environmental harshness or poor physical condition impacting the reproductive system^2,5–11^. Ecosystems, and particularly the marine environment, are rapidly changing due to broad-scale natural and anthropogenic processes^12–14^. Consequently, marine mammals are increasingly exposed to a variety of stressors, including chemical and noise pollutants caused by e.g., ship traffic, seismic surveys and construction, as well as marine litter, disturbance, habitat loss and loss of prey through competition with fisheries^15–22^.

Harbour porpoises (*Phocoena phocoena*) are K-strategists with their reproduction characterized by 10–11 months of gestation of a single offspring^23^. If conditions are good, porpoises may mature at 3–4 years of age and produce a calf every year thereafter^24,25^. They inhabit cold temperate to sub-polar waters of the Northern Hemisphere and are one of the most abundant cetaceans^26,27^. Due to their small size, limited capacity for storing energy and their high metabolic rate, porpoises must eat large quantities of high energy prey to sustain themselves^8,20^. They feed on a variety of prey and diets differ considerably across their range^28,29^. It is therefore conceivable that regional differences in prey and habitat quality, as well as in anthropogenic activities, affect their health and subsequently their reproductive capacity, with cascading effects on population vital rates. Studies on reproductive impairment in harbour porpoises have predominantly been focussed on associations with PCB exposure^16,17,30–33^, while studies on the relative importance of other causes remain scarce.

Here, we present a comprehensive analysis into factors driving reproductive success of harbour porpoises. We investigate whether reproductive females abandon investment in their foetus when intrinsic or extrinsic conditions are poor, presumably to prioritize their own survival and thus long-term fitness. To study the role of intrinsic conditions, data on health, cause of death, fat and muscle mass, and diet of porpoises from Dutch waters were categorized and analysed. We expect a lower pregnancy rate (PR) in females in poor nutritional and health status compared to those in good nutritional and health status, and a positive relationship between foetal size and nutritional status, based on blubber thickness, visceral fat and muscle mass of the mother. These results were used in the second part of this study, in which we examined the role of extrinsic conditions on life history parameters. We compiled records of PR and age at sexual maturity (ASM) from 17 study areas across the distributional range of the harbour porpoise. For each area, we established proxies of environmental condition: mean energy density of prey that constituted porpoise diet (MEDD) which is a weighted average energy density of all consumed prey at a given location, cumulative human impact (CHI) based on a model containing data on climate change, fishing, land-based pressures, and other commercial activities^12^, and reported levels of polychlorinated biphenyl (PCB) contamination^17,34^, with the aim of determining which of these environmental condition(s) best explain reported life history parameters.

## Results

### Dutch waters: foetus growth

**Figure 1:**
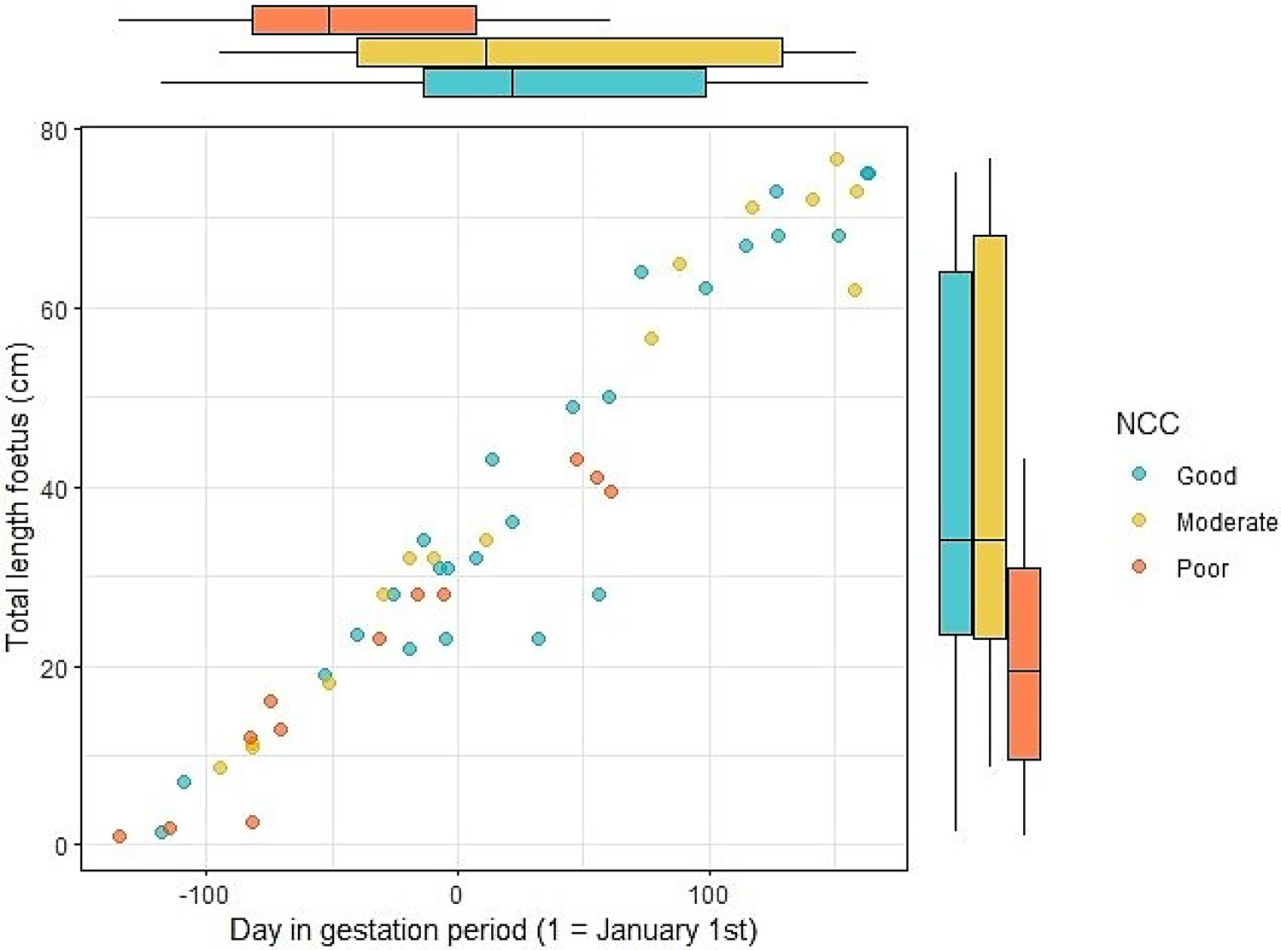
Foetus length as a function of nutritional condition of the mother. Combined scatterplot and boxplots of foetus length (y-axis) throughout the gestation period (x-axis). Coloured by nutritional condition category (NCC, based on blubber thickness, visceral fat and muscle mass assessed during post-mortem examination) of the mother (n = 50), with blue = good, yellow = moderate and orange = poor. One pregnant female carrying a twin was excluded.

Between 2006 and 2019, 199 mature female harbour porpoises were necropsied and a total of 52 foetuses were found: 50 singular and one twin. The smallest recorded foetus was approximately 10 mm, found August 18th, and the largest foetus was 76.5 cm, found on the 31st of May (SFig. 1). In order to identify which parameters influence foetus size, we assessed the effect of Julian date, to account for foetus growth which increases throughout gestation, the effect of total length of the mother, her health status (based on her cause of death), her nutritional status (firstly based on blubber thickness corrected for season (corBT) and secondly based on a nutritional condition code (NCC), taken into account blubber thickness and assessment of visceral fat and muscle mass), as well as interactions between these parameters (Model 3 with corBT and Model 4 with NCC, see methods for full description). Foetus size was mostly influenced by Julian date (proxy for day in the gestation) and by the nutritional status of the mother, but not by the length of the mother or her health status. This is indicative of strong positive relationships: increasing day in gestation increased foetus size, and additionally, an increasing nutritional status of the mother was positively correlated to an increased foetus size (Fig. 1, SFig. 2). No interactions between the predictor variables were retained in the final model. The lack of a significant interaction between Julian date and nutritional status indicates that the effect of nutritional status of the mother on foetus length remained the same throughout the study period.

### Dutch waters: pregnancy rate

Excluding all mature females from the months May–August, to reduce the error of missing pregnancies as calving and conception occurs in these months, there were 119 mature females of which 41 were pregnant, giving a PR of 0.34 (95% CI: 0.26–0.43). Including the conception period (May–August) revealed a PR of 0.28 based on 51 pregnancies among 180 mature females (95% CI: 0.22–0.35). The PR for mature females in the health category which included mature females dying as a direct result of anthropogenic trauma or predation was higher: 0.58 (95% CI: 0.41–0.74, excluding conception period) based on 22 pregnancies among 38 mature females. The PR for mature females in the health category which included porpoises dying due to infectious disease or emaciation was lower: 0.24 (95% CI: 0.14–0.34, excluding conception period), based on 17 pregnancies among 71 mature females.

To test which variables influence pregnancy among the mature females, we assessed the effects of age, year, month, health status, nutritional status and several interactions among these variables (Model 5 with corBT and Model 6 with NCC, see methods for full description). Pregnancy was best explained by health status, and not by any of the other predictor variables or interactions between them (log OR [95% CI] = − 1.79[− 3.07 to 0.66] for factor(Health)debilitated). OR was 0.17 (95% CI: 0.05–0.52), demonstrating that the odds of being pregnant reduce when the female is in a debilitated health status.

### Dutch waters: prey energy density

Mean energy density of prey constituting porpoise diet (MEDD) was calculated for the entire dataset [all necropsied porpoises examined in The Netherlands with food remains in their stomachs (n = 985)] and for various sub-sets of these data (Table 1). MEDD at large was relatively low (Table 1; < 5.45 kJ/g) and we found little or no difference between MEDD of adults and immatures, between adult females and adult males, or between pregnant and non-pregnant (adult) females. However, MEDD of mature females in the health category which included porpoises dying as a direct result of anthropogenic trauma or predation was on average 16% higher than the MEDD of mature females in the health category which included mature females dying due to infectious disease and/or due to emaciation of unknown origin. In addition, MEDD of mature females in good nutritional condition was also higher compared to mature females in moderate or poor nutritional condition (Table 1).

**Table 1:** Mean energy density of prey constituting porpoise diets (kJ/g) (MEDD) of the full dataset (all harbour porpoises from Dutch waters (new) (All HP-NL)) and for various sub-sets of animals.

Category	N	MEDD
All HP-NL	985	4.92
All adult	280	4.87
All immatures	702	4.97
Adult females	171	4.83
Adult males	110	4.88
Adult females-pregnant	45	4.76
Adult females-not pregnant	96	4.77
Adult females dying acutely, e.g. due to anthropogenic trauma or predation	55	5.06
Adult females dying as a result of infectious disease and/or emaciation	79	4.37
Adult females in good NCC	56	5.45
Adult females in moderate NCC	69	4.49
Adult females in poor NCC	58	4.47

N-values give the numbers of animals with food remains in their stomachs. NCC = nutritional condition category, based on assessment of blubber thickness, visceral fat and muscle mass during post-mortem examination.

### Dutch waters: age at sexual maturity

ASM of female porpoises found dead in The Netherlands was calculated from established ages of 154 individuals, comprising 32 immature and 122 mature females. The oldest immature female was 5.5 years, and the youngest mature female 3 years of age. ASM was calculated at 4.0 years (95% CI: 3.47–4.48 years, Model 7, SFig. 3). The maximum age at death was 24 years, however, the median age at death was 8 years, with a mean of 7.9 years and third quartile of 8.5 years, revealing that 75% of the mature females did not live longer than 8.5 years.

### Global: life history parameters and environmental conditions

Nineteen studies were found that reported PR and sixteen that reported ASM for harbour porpoises, including the results of this study (STab. 2, the current study is referred to as ‘Dutch waters—new’). PR ranged from a minimum of 0.28 in the Salish Sea to a maximum of 0.99 in Icelandic waters (STab. 3). ASM ranged from 3.1 in Eastern-Newfoundland to 5.5 years off the North-West Iberian Peninsula (STab. 4). MEDD and cumulative human impact (CHI) scores could be calculated for 17 of the study areas, PCBs (sum of all) for seventeen, of which thirteen remained when restricting the analysis to ≥ ∑17PCB–≤∑99PCB (Table 2, STab. 12). An overview of these data is provided Table 2, where the study areas are organized by the lowest to highest PR with associated data on ASM, MEDD, CHI, and contaminant scores.

**Table 2:**
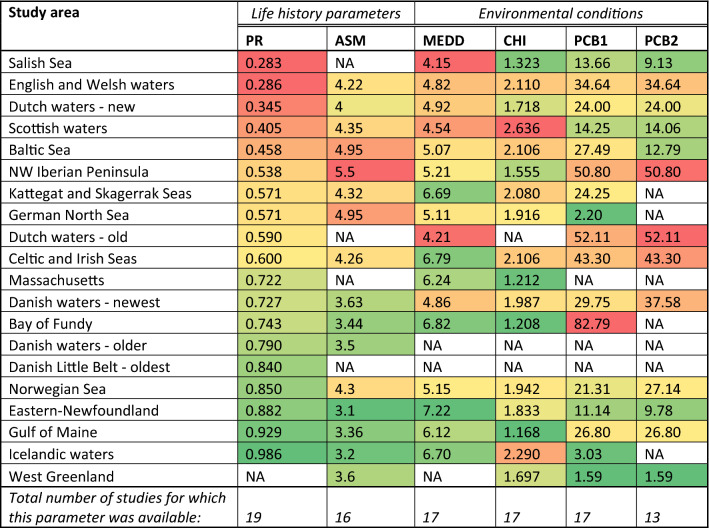
Summary table of pregnancy rates (PR), age at sexual maturity (ASM), mean energy density of diet (MEDD), cumulative human impact (CHI) mean scores, and chemical pollution by PCBs (mg/kg lipid weight) of harbour porpoises in study areas across their distributional range.

Note that PCB is given twice; PCB1 is without restrictions on the sum of congeners and mixtures reported, and PCB2 is restricted to studies reporting ≥ ∑17PCB− ≤ ∑99PCB. The table is organized by study area arranged from lowest to highest PR with colour shadings corresponding to lowest (reddest) to highest (greenest) PR. All entries of the other columns are coloured as highest (reddest) to lowest (greenest). Time frames of studies and references are given in STab. 2.

The MEDDs ranged from 4.15 to 7.22 kJ·g^−1^ with a global average of 5.51 (SD: 0.96) kJ·g^−1^ (STabs. 5–9). All diet studies reported various prey species, but in each study, only a few prey species contributed substantially to the energy density of the diet. For study areas with a high MEDD there was a dominance of high-energy density prey (generally herring) while study areas with lower MEDD values showed a higher abundance of low-energy density prey (mostly *Gadidae* spp. like cod and whiting) (STab. 5–6). Although this geographical variation was evident, some prey species or prey groups were found to be included in the diet of porpoises at nearly all study sites: gadoids were found in all (17/17) studies, clupeids in 16/17, sandeels in 15/17, squid in 12/17 and gobies in 11/17.

According to previously published metrics of cumulative human impacts of world oceans^12–14^, North-west European waters had the highest maximum CHI scores, with maximum scores of 7.91–8.39 in the German, Dutch and English North Sea as well as the Norwegian Sea, but the mean score was highest for Scottish waters (CHI mean score of 2.62). Waters around Canada and the US generally showed lower mean CHI scores compared to European waters (STab. 10).

PCB measures where available for a total of 484 individual adult male harbour porpoises, which are considered to be a better proxy to measure area specific environmental pollution than females or porpoises in other age classes^34,35^. Data was available from varying numbers per area of which the life history parameters were gained, ranging from only n = 1 for the NW Iberian Peninsula (∑PCB of 50.8) to the maximum of n = 127 for Scottish waters (mean ∑PCB of 14.47 with SD: 12.04). Lowest ∑PCBs were reported for West Greenland (∑PCB of 1.59), the German North Sea (∑PCB of 2.2) and Iceland (∑PCB of 3.02) and highest for the Bay of Fundy (∑PCB of 82.79). The number of congeners included by the different studies varied additionally, and therefore the analyses were conducted on two subsets of data: PCB1 refers to all studies, whilst for PCB2 the analyses were restricted to studies reporting ≥ ∑17PCB–≤∑99PCB (Table 2). Lowest ∑PCBs were found for West Greenland (∑PCB 1.59) the Salish Sea (∑PCB of 9.13), and Eastern-Newfoundland (∑PCB of 9.78), and highest for the water around the NW Iberian Peninsula (∑PCB of 50.8) and the older Dutch study (∑PCB of 52.11) when the analysis was restricted to ≥ ∑17PCB −  ≤ ∑99PCB (STabs. 11 and 12).

**Figure 2:**
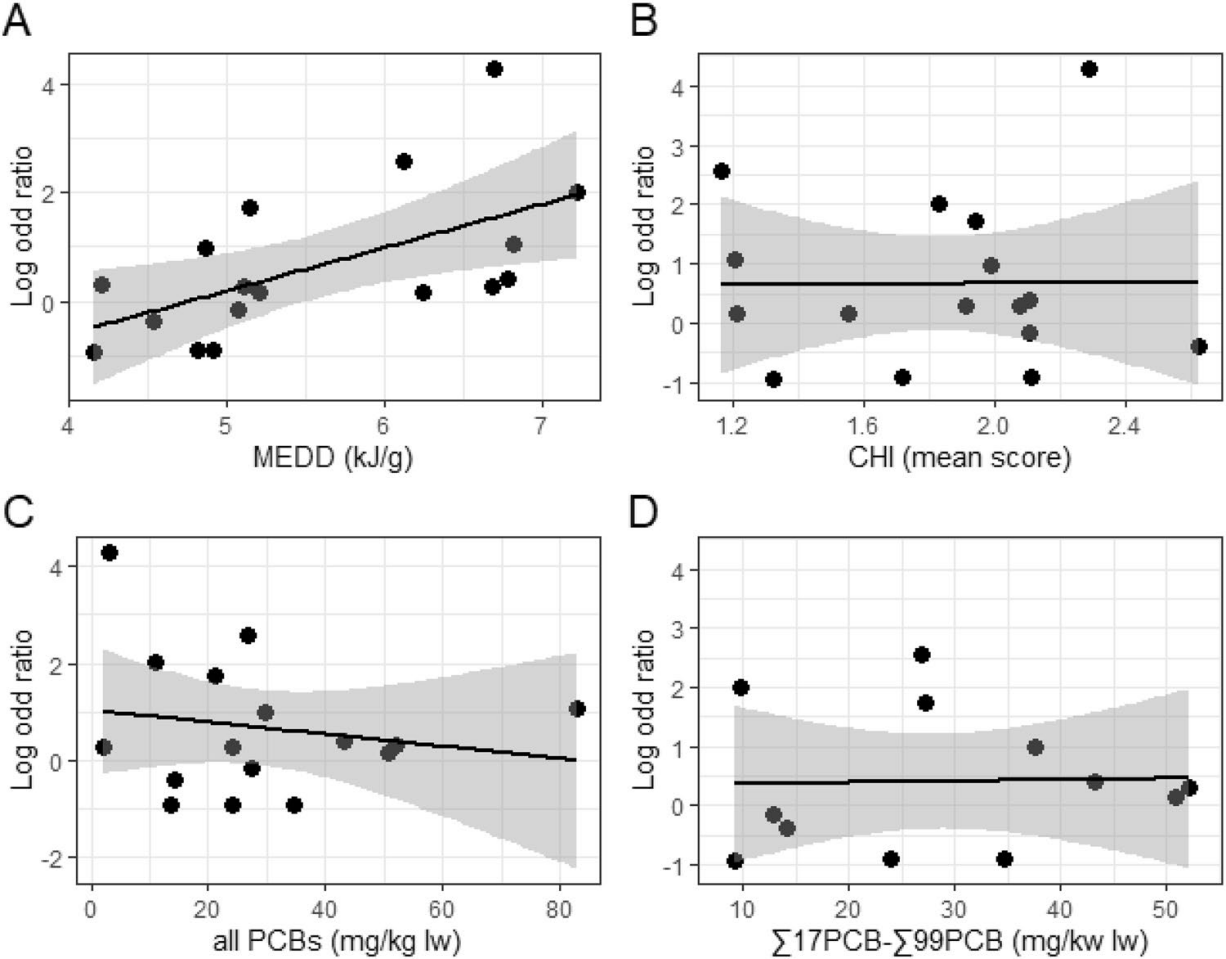
Log odds ratios (y-axis) of being pregnant (versus being non-pregnant) in relation to the environmental conditions (x-axis): **(A)** the mean energy density of the diet (MEDD) (STab. 5), **(B)** cumulative human impact (CHI) mean scores (STab. 10), **(C)** chemical pollution by PCBs (PCB1) and **(D)** chemical pollution by PCBs restricted to studies reporting ≥ ∑17PCB− ≤ ∑99PCB (PCB2) (STab. 11 and 12). A linear regression line is fitted to all graphs, with the grey shaded areas reflecting 95% CI.

The model which best explained the number of pregnant females (N*preg*) in the total number of females (Ntotal) had both MEDD and PCB1 as the predictor variables, and not CHI. The confidence intervals for the estimated log-OR of this model revealed a strong, positive association with MEDD (log-OR[CI95%]: 0.98[0.46–1.51]), and a weaker, negative association with PCB1 (log-OR[CI 95%]: − 0.02[− 0.04 to 0]) (Model 8, Fig. 2, SFig. 4). When replacing PCB1 by PCB2 (thereby reducing the number of study areas), only MEDD was retained in the final model.

None of the environmental parameters assessed here explained the variance in ASM between the study areas (Model 9). However, the number of observations (the N *total* per study area), which was used to weight the model, varied greatly between study areas (range n = 32–354). The square root of the residual deviance divided by the degrees of freedom was large (6.29 for the model with PCB1 and 5.17 for the model with PCB2) and findings therefore likely dominated by the difference in sample size per study area.

## Discussion

In mammals at large, maternal malnutrition has been shown to compromise conception, length of gestation, and foetal growth and development, with poor maternal health also affecting the onset of sexual maturity, and leading to pregnancy termination, preterm deliveries, small birth sizes and birth defects^4,9,10,36–39^. Our study showed, both at a local scale and globally, that prey energy density, health status and nutritional status contribute importantly to reproductive success of harbour porpoises. In our local study population (Dutch waters) we demonstrated that maternal nutritional and health status affect foetus size (Fig. 1) and the probability of being pregnant, respectively. Globally, pregnancy rates were best explained by the energy density of prey eaten and, to a lesser extent, by pollution with PCBs (Fig. 2, SFig. 4). High-energy prey is probably crucial for maintaining a good health status for porpoises^8,40^, and particularly for mature females if they are to reproduce. Separating mature porpoises by their nutritional status (Table 1) showed that animals in good condition had a much higher mean energy density of prey in their diet than those in moderate or poor nutritional status. It remains unclear, however, whether a high-energy diet led to a good condition or whether only animals in good condition are able to catch and handle high-energy prey. Some high-energy prey might be relatively hard to catch, as these live buried in the sediment (sandeels), or are fast swimmers (mackerel). Large prey with a high energy may also be dangerous to swallow^40^. Catching high-energy prey may thus require special skills or take considerably more effort. Predators have to develop and adapt their foraging strategy to their energy requirements^8,41^. These requirements increase significantly when mature female porpoises become pregnant or start lactating. While mature harbour porpoises are clearly able to increase the energy density of their diets (Table 1), it remains uncertain if individuals with a compromised health or nutritional status can maintain a high-energy diet. Thus, pregnant females may be forced to abort their foetus if intrinsic or extrinsic conditions deteriorate and their own survival is at stake.

Reproductive impairment has previously predominantly been associated with PCBs, with numerous studies describing negative effects on marine mammal health. This includes implantation failure, abortion and sterility in pinnipeds^18,42,43^, and increased first-born calf mortality, as well as infectious disease and the development of cancer in cetaceans^43–46^. Despite this, in our study, ambient PCB levels did not associate well with porpoise pregnancy rates and age at sexual maturity. This could be a result of the discrepancies between the samples used in previous studies compared to the PCB proxy used here; PCB levels measured in adult male porpoises as proxy of environmental contamination. This data was more abundantly available in literature and it reduced the bias of offloading which occurs in mature females^34,47^, thereby providing a more accurate representation of area specific maximum levels of contaminants. It is however possible that PCB levels measured in males, as well as the two life history parameters assessed here are (in combination) not suitable as biomarkers for the assessment of population effects of PCB pollution on cetaceans. We were additionally challenged by the many differences in PCB congeners examined, analytical protocols used between studies, and the level of detail at which the data were reported. Restricting to more similar congener groups analysed (≥ ∑17PCB–≤ ∑99PCB) significantly reduced the sample size. Besides, data on other pollutants which may severely affect marine wildlife^48^ was not available. This highlights the need for more consistent, long-term, global contamination data measured in the same tissues with similar calibrated techniques. Also, other approaches or biomarkers to assess pollution impact on small cetaceans should be established or further explored, e.g., through using individual-based model frameworks^34,43,49,50^.

The increasing exposure of marine mammals to multiple stressors such as ambient noise and climate change is stated to be a major knowledge gap in marine ecology^22,51–54^. The assessment of individual pressures as well as the cumulative effects of multiple stressors on marine mammal populations is highly challenging and a way of quantitatively assessing these effects is yet to be established^53,54^. We explored the ecosystem-specific, multiscale spatial model by Halpern et al.^12–14^ and used this as a proxy of CHI on the study areas. Using this validated and published model had the advantage that CHI scores could be assessed equally for each study area, which was contrasted with the diet and PCB parameters for which it was unavoidable to use studies with different sample sizes, methodologies or spatiotemporal mismatches (STab. 2). The CHI model was however gained from a relatively recent study^13,14^, whilst the life history studies were often conducted over several years and could also include data from previous decades (STab. 2). Additionally, the CHI model did not necessarily consider the stressors which directly apply to or specifically affect small cetaceans, since it was built to quantify the effects of human activities on the world’s oceans. The CHI scores were therefore a measure of stressors affecting the environment and although it is likely that negative effects on oceans directly or indirectly translate to the top predators living there^55^, our analyses did not identify a direct negative association with the crucial life history parameters assessed. However, from a porpoise’s perspective, the resolution for the CHI assessment used here are rather crude. This can be particularly true for areas where local, emerging, or natural threats affect cetacean populations. Examples are the Baltic and North Seas, where there is large scale construction of offshore wind farms^56,57^ as well as predatory pressure from grey seals (*Halichoerus grypus*) on porpoise populations^58^. It is therefore recommended to add local threat layers, as well as to assess individual threat layers apart from a cumulative approach, before drawing smaller-scale or regional conclusions on the effects of anthropogenic stressors on cetaceans.

Our results present the current best overview of life history parameters of porpoise populations throughout their range, based on a long-term, large dataset containing information from stranded and bycaught individuals. Although there is uncertainty to which extent data from deceased cetaceans are representative of the at-sea population, it is unavoidable to use this, as longitudinal or alternative methods to determine reproductive parameters are currently lacking^53,54,59–61^. Pregnancy rates and MEDD differed between porpoises in the different health and nutritional categories, as demonstrated both in our sampling population (Dutch waters) (SFig. 2, Table 1) and in the global assessment. Globally, the highest pregnancy rates were reported in studies using specimens gained from fisheries (STab. 3). Results from studies on bycaught porpoises intrinsically differ from those based on stranded individuals. The latter constitute a mixture of animals dying of disease, bycatch, or e.g., interspecific interactions. Yet, regardless of an individual’s eventual cause of death, a reliable assessment of health status should be conducted through extensive pathological assessment, since bycaught marine mammals may also display significant disease or poor nutritional conditions^62,63^. Therefore, for future studies, both at global and local scales, it is vital to examine and report, in concert, pathology, ecology, toxicology and life history in order to estimate and correct for the effects of health, nutritional or confounding and interacting factors on life history parameters.

We assessed prey quality, expressed as mean energy density, as a determinant of reproductive success. Previous studies on a range of piscivorous top predators suggested that a shift towards low-quality prey, regardless of an availability of high-quality prey, may have detrimental effects on populations^9,64–66^. Abundantly available low-quality prey that might be easily caught, such as gobies or small gadoids, may thus act as an ecological trap for porpoises, that should probably better pursue prey of higher energy density, even if these are harder to catch. Quantifying the effects of prey choice on the predators’ vital rates requires knowledge on population development, food-web structures, predator energetic requirements and prey energy densities; all of which may vary seasonally, geographically or temporally, both for the predators and for their prey^41,67,68^. Studying the effect of nutritional stress in free-ranging animals is therefore complex and requires the understanding of many factors^68^. We did not assess prey availability, since this data is largely lacking for many of the non-commercial fish species which are part of porpoise diets, but we were able to establish proxies of prey energy density for all but one of the study areas. It should however be kept in mind that we had to use mean prey energy densities, as well as mean CHI and PCB scores, since the input data each had their own limits and uncertainties. Modelling of mean values and thus data summaries per study area were conducted using a random area effect to at least partially account for unknown and unmeasured factors in which areas may differ, but these approaches can result in hidden correlations or incalculable biases. Despite that there are still many uncertainties regarding the direct and indirect causes and consequences of nutritional stress on cetaceans, we here found a strong association between prey energy density and pregnancy, based on large numbers of animals and studies. The present study advances our understanding of risks that may affect harbour porpoise life history traits. We show the necessity of not focussing solely on chemical pollution when assessing reproductive impairment, but to incorporate different and cumulative factors, and, in particular, we highlight the access to undisturbed prey with high energy density as a crucial factor for reproductive success.

## Methods

### Data collection and preparation—Dutch waters

#### Study specimens

Between 2006 and 2019, 1457 deceased harbour porpoises in The Netherlands were collected for post-mortem investigations and diet analysis, with the necropsies conducted following internationally standardized guidelines^69^. For this study focussing on female life history, we selected all females > 115 cm, as smaller animals may be maternally dependent and can be considered young of the year^23^. Cases in DCC5, which represent carcass remains, and of which reproductive organs were not assessable or present due to scavenging or incompleteness of the carcasses were excluded. The reproductive organs of the female porpoises > 115 cm (n = 328/1457) were macroscopically inspected to differentiate between immature and mature animals, with the presence of ovarian corporal scars used as indication of maturity^70^. Exact age was determined by assessing tooth growth layer groups (GLG) for a subsample of cases (n = 154), according to previously described methods^71^. Data can be found in STab. 13.

#### Health and nutritional status

Porpoises collected for post-mortem investigation were necropsied with the primary aim to determine the animals’ causes of death and their health status, with the quantity and quality of data and results strongly depending on carcass freshness and completeness as well as other logistical and financial factors^69^. For this study, we established three proxies based on the findings and metrics taken and assessed at necropsy: a proxy for health status and two proxies for nutritional status. For the proxy for health status, we assessed the cause of death among the mature females (n = 199/328) and divided all cases in two categories. In the first category, we placed all mature females which most likely died as a direct result of incidental bycatch (diagnosed based on the presence of encircling imprints or external incisions, the recent ingestion of prey, and the exclusion of other causes of death, for more details see IJsseldijk et al.^63^), as a direct result of a predatory attack (diagnosed based on the presence of large, sharp-edged mutilations with associated ante-mortem bite lesions bilaterally on the tailstock, extremities or on the head, for more details see Leopold et al.^58^) or as a direct result of another acute cause, such as sharp forced trauma or dystocia (obstructed labour, full-term foetus) which did not present signs of significant disease or debilitation. All other animals were placed in the second category, with these mature females displaying evidence of general and significant debilitation, including infectious disease (such as significant parasitism, bacterial, viral or mycotic infections) and/or emaciation. Cases that could not be grouped, mostly as a result of decomposition, were excluded from analyses that included this as a parameter.

The first proxy of nutritional status was based on the mean blubber thickness, measured during necropsies in a dorsoventral line on the left body flank just cranial to the dorsal fin, at three locations: dorsal, lateral, and ventral. Blubber thickness in small cetaceans has previously been shown to decrease during periods of fasting^72,73^ and this metric has been used as proxy of nutrition by others^45,74–76^. However, it should be noted that blubber thickness is not always a good reflection of individual health nor cause of death (e.g., animals dying of acute causes could also be debilitated^62,63^). There is uncertainty to what extent factors such as age, sex and season naturally influence blubber thickness, and this should be accounted for. Since we focus our analyses on mature females, no further correction for age and sex was done. However, to correct for season, we modelled the mean blubber thickness as a function of Julian date using a generalized additive model (GAM) to allow a smooth effect of the predictor variable (Julian date). This captures the sinus-shaped seasonal variation in blubber thickness which naturally occurs as a result of changing water- and air temperature^25,72^ (SFig. 5). The residuals of that model were thereby indicative of an adult females’ nutritional status independent of season, and hence they were used as the proxy for nutrition (referred to in the main text as: nutritional status using corBT, Model 1 in STab. 1).

The second proxy of nutritional status used the categorical variable “nutritional condition” (NCC), which is assigned during necropsies as good, moderate, or poor. Animals in good NCC generally presented a convex outline on a cranial perspective, no signs of muscle atrophy (abundant skeletal musculature) and presented signs of visceral fat. Animals in moderate NCC generally did not have a fully round outline on a cranial perspective, showed possible signs of muscle atrophy and did not present visceral fat. Animals in poor NCC generally had a concave outline on cranial perspective, with visible aspects of vertebrae and/or scapula externally, an hollow appearance caudal to the skull and signs of muscle atrophy (based on IJsseldijk et al.^69^). Since this categorial differentiation is collinear with the first established proxy of nutritional status (SFig. 5), it was not used in the same modelling procedures. Therefore, models were run twice, first with corBT and secondly with NCC (for an overview see STab. 1).

#### Pregnancy rate and foetus size

The pregnancy rate (PR) was calculated as the proportion of pregnant females in the total sample of mature females (following e.g.,^70,77^). Pregnancy rates were also calculated separately for the animals in the two different health status categories (see above). To avoid missing the presence of very small, early embryos, samples from the period of conception (June–August^23^) as well as samples from the period of calving (May–June^23^) were excluded in the PR calculations. All foetuses were measured during necropsy (of the dam) and a proportion of these were also weighed.

#### Mean energetic density of diets

As a measure of the quality of prey species constituting the diet of harbour porpoises necropsied in The Netherlands, we calculated the mean energy density of their diet (MEDD). Prey were identified from stomach contents, mostly from otoliths; for each individual prey that could be identified, the fresh mass was estimated (using^78^ and following^29^) The energy density (ED) is defined as the energy per kilogram of wet weight of prey^8,79^. ED values for all prey species encountered were taken from the literature (STab. 7). If for a given prey species no value for ED could be found, the ED of a comparable species (mostly same genus), or the mean value of its family, was used. For species for which multiple ED values were available, values were averaged. ED values reported in kcal were multiplied by 4.184 to convert to kJ (following e.g.^80^). To calculate the mean ED of the diet for a group of porpoises (MEDD, kJ·g^−1^, see Table 1) we used:

1 $$MEDD=\frac{1}{\sum_{i=1}^{n}{M}_{i}}\sum_{i=1}^{n}({M}_{i}*{ED}_{i})$$

where *i* is the prey species and *M* the reconstructed prey mass in grams (following^8^). The reconstructed prey mass per species is multiplied by the species-specific ED and the energy sum is divided by the total mass of all prey, resulting in the MEDD.

### Data analyses and statistical models—Dutch waters

Data were explored prior to analyses following Zuur et al.^81,82^. Data exploration and analyses were performed using R version 3.6.3^83^, with packages ggplot2, grid, gridExtra, rsq, glmTMB, mgcv and ggpubr. Several statistical models were developed (for referencing in the text see overview in: STab. 1).

#### Influences on foetus size

To identify which variables influence foetus size, we firstly identified the best measure for foetus size. A Generalized Linear Model (GLM) for foetus length and weight was fitted (Model 2), with weight only available for a subset of all foetuses (n = 34). This model indicated a close relationship between length and weight (R^2^ of 0.8 for foetus length as a function of mass, SFig. 5), and foetus length was therefore used as representative for foetus size in the subsequent analysis, to increase sample size. GLMs with a Gaussian distribution were used (Model 3). The model selection tested for covariates and their influence on foetus length, with the predictor variables: Julian date to account for foetus length which increases throughout gestation, total length of the mother, health status of the mother, nutritional status of the mother. Interactions between length of the mother and her nutritional status were included following data exploration. Only cases with complete observation of all parameters were included (n = 43). A backwards model selection approach was applied with the drop1 function from the R language used to assess which model terms could be excluded^83^. The best fitting model was selected using Akaike’s Information Criterion (AIC), which provides a relative measure of the goodness of fit of statistical models. Model validation was done to identify potential violations of model assumptions by inspection of normalized residuals and assessment of residual probability plots. Likelihood profile confidence intervals (95%) and odds ratios of the most optimal model were calculated. Models were run twice, first using the first proxy of nutritional status based on blubber thickness corrected for season (corBT) and secondly using the nutritional condition category (NCC), taken into account blubber thickness, visceral fat and muscle mass (for full descriptions, see above).

#### Influences on pregnancy

To identify which variables influence pregnancy, we firstly coded all mature, pregnant females as 1 and all mature, non-pregnant females as 0. Next, GLMs with a binomial error distribution and logit link were used (Model 4) to test the influence of included covariates on the likelihood of pregnancy. Only cases with complete observations of all parameters were included (n = 65). The predictor variables included in the saturated model were age, year to assess temporal variance, month to assess seasonal variance, health status (proxy, categorical), and nutritional status. Interactions were added following data exploration: between health and nutritional status, between the health status and year and health status and month. Model selection, validation and interpretation was conducted following the protocol previously described above. Models were run twice, first using the first proxy of nutritional status based on for season corrected blubber thickness (corBT, numerical) and secondly using the nutritional condition category, taking into account blubber thickness, visceral fat and muscle mass (NCC, categorical) (for full descriptions, see above).

#### Age at sexual maturity

The age at sexual maturity (ASM), or age at 50% maturity, was determined using binomial logistic regression models. Maturity, coded as 1 for mature females and 0 for immature females, was modelled as a function of age (in years) to assess ASM (n = 154, Model 5). The model was fitted using a binomial error distribution and logit link, as is appropriate for binary data and the ASM was estimated by calculating the negative of the slope over the intercept.

### Assessment of porpoise life history and environmental condition globally

The life history response variables assessed were PR and ASM, which were obtained from 17 different studies. The earliest study was conducted between 1941 and 1943, but the majority of the studies were performed between 1980 and 2019 (including the present study). The environmental predictor variables used were quality of diet, expressed as mean energy density of diet (MEDD), cumulative human impact (CHI) with data on climate change, fishing, land-based pressures, and other human activities, and lastly chemical pollution expressed as polychlorinated biphenyls (PCBs). Fifteen diet studies were used, ranging from 1985 to 2019 (including this study). One comprehensive study was used to obtain the CHI information for the year 2008. A total of 21 studies reporting PCB levels in harbour porpoises, conducted in the period 1971–2019 (including the present study) were collated. Details below and in STab. 1.

#### Life history

For PR the following were tabulated: (1) the number of pregnant females out of the total number of mature females in each study, (2) the determined conception period and whether this was accounted for in the calculation of the PR, (3) the method to assess pregnancy, which was either based on the presence of a foetus or presence of a corpora lutea (CL), and (4) the source of the specimens: either directly from fisheries, strandings including trauma cases, or a combination thereof (STab. 3). For ASM we provide: (1) how ASM was assessed in each study, and (2) the standard error (SE) or confidence interval (CI), if reported (STab. 4).

#### Energy density of prey

A literature search was performed for diet studies from stomach contents of porpoises from or near the study areas where PR and ASM were determined. When multiple diet studies were available the study was selected that best corresponded to the time frame at which PR and ASM were calculated. For the diet studies which reported the reconstructed prey mass in grams we used formula (1) (STab. 8). When the prey mass was reported as a percentage of relative abundance in terms of estimated biomass of prey (%M), we multiplied %M by the ED of the prey species and divided the total %M (STab. 9), using:

2 $$MEDD=\frac{1}{\sum_{i=1}^{n}{\mathrm{\%}M}_{i}}\sum_{i=1}^{n}({\mathrm{\%}M}_{i}*{ED}_{i})$$

For the studies where the %M was presented in a bar chart, we measured the %M using digital callipers.

#### Cumulative human impact

An ecosystem-specific, multiscale spatial model containing high resolution data on the intensity of human stressors and their impact on marine ecosystems was developed by Halpern et al.^12,14^ as part of their Ocean Health Index project. CHI values derived by this model are based on fourteen stressors related to human activities from four primary categories: (1) land-based drivers, including nutrient pollution runoff, organic chemical pollution runoff (pesticides), direct impact of humans (density of coastal human populations), and light; (2) five types of (commercial) fishing, including commercial demersal destructive, commercial demersal non-destructive high bycatch, commercial demersal non-destructive low bycatch, pelagic high bycatch, pelagic low bycatch, and artisanal; (3) climate change, including sea surface temperature, ocean acidification and sea level rise; and (4) shipping. Extensive descriptions of these drivers are published in the methods and supplementary material of Halpern et al.^12,14^, including information on the origin and validation of the data.

For this study we used the global CHI dataset that is publicly available via the Knowledge Network for Biocomplexity^14^. Data on CHI was based on the year 2008. We extracted the CHI scores for each of our study areas at ~ 1 km^2^ resolution and calculated the min, max, mean and median values. To do so, we defined our study areas using the standard georeferenced marine regions as published under the Flanders Marine Institute^84^. In most cases we combined two or more regions from the database to get full coverage of the study area, but for the study areas where the marine regions did not provide full coverage, we used a manually created polygon. The list of regions is given in STab. 15. For areas with more than one life history study (Denmark and The Netherlands) we used the newest studies since these provided the better match to the time of the CHI score calculation (STab. 2).

#### Chemical pollution

Polychlorinated biphenyls were not included in the list of organic polluters by Halpern et al.^14^. However, PCBs have been specifically associated with reproductive impairment in many marine mammal species^16–18,30,50^, therefore the correlation with life history parameters for this industrial organic pollutant was assessed separately. Data was retrieved from the International Whaling Commission’s (IWC) ‘POP Contaminants Trend Explorer’ tool, hosted on the portal of the Sea Mammal Research Unit (SMRU, University of St. Andrews, Scotland). This tool is established under the IWC Scientific Sub-Committee on Environmental Concerns (IWC SC/68A 2019) as part of the IWC Pollution 2020 Initiative and includes data from scientific publications from the 1970s–2000s^34,43^. The database was provided by the tool manager and included data restricted to adult males, to reduce the bias of biotransfer of chemicals, which occurs during gestation and lactation in females^35^. The tool reports PCB concentrations in blubber, which is the most commonly assessed tissue in marine mammals for studying the burden of the highly lipophilic and stable PCB compounds^47^. PCB concentrations that were measured in porpoises in the same areas from which life history parameters were verified with the literature and included. In addition, the literature was searched for PCB analyses of harbour porpoises published in the 2010s, as well as own institutional databases, and data added to align time frame, where possible, with time frame of conducted life history studies (STab. 2).

The presentation of concentrations of pollutants was based on either wet weight (ww) or lipid weight (lw). To allow comparison, the datapoints need to be converted to one common unit, with lw most frequently reported. Studies reporting only ww or dry weight were not included. Studies reporting ww and percentage of lipids (%lipids) were converted to lw, using:

3 $$lw=\frac{ww}{\%lipids}*100$$

The datapoints were converted to mg/kg lw for all studies and the mean ∑TotalPCB is reported per area.

The variance of the sum of congeners reported ranged from ∑6PCBs up to ∑99PCBs, with several older studies reported Aroclor mixtures. Data per congener was however largely not available in literature. We therefore present two mean ∑PCB datapoints: firstly including all studies regardless of the sum of congeners or mixtures (referred to as PCB1), and secondly limited to studies reporting ∑17-99PCBs (referred to as PCB2).

#### Statistical models for global assessment

For the analyses we restricted to study areas with complete observations of the environmental conditions to compare models. A GLM fitted with a binomial distribution and logit link was used to determine the effect of environmental conditions on pregnancy rates (Model 6). The response variable was the number of pregnant females (N*preg*) in the total number of females (N*total*) (grouped binomial data, STab. 3). Since the differences between study areas can be large because of unknown effects, an individual normal random effect for area was added on the logit scale. Another GLM was conducted to determine the effect of the three environmental conditions on age at sexual maturity (Model 7) fitted with a Gaussian distribution and weighed by sample size (N*tota*l) (STab. 4). This model was applied twice using two individual predictor functions: first, with the predictor variables MEDD, CHI and PCB1 and secondly with the predictor variables MEDD, CHI and PCB2. The latter restricted the analyses to a smaller number of study areas due to missing data, but it reduced some of the bias because of very small (< ∑17PCBs) or very large (Aroclor mixture) reported ∑PCB datapoints.

For all models, a backward stepwise model selection process using the drop1 function was conducted and the best models explaining the life history parameters pregnancy rate (Model 8) and age at sexual maturity (Model 9) were identified by assessment of the AIC. The logistic regression model yields log-odds ratios. This represents the odds (number of pregnant per non-pregnant) of being pregnant compared to non-pregnant in each study area, while accounting for differences in sample size. We also obtained the confidence intervals for the estimated log-odds ratios of the most optimal models.

### Ethics statement

The animals described in this study were free-living harbour porpoises which died of natural causes. No consent from an Animal Use Committee is required, as the animals described in this study were not used for scientific or commercial testing. Consequently, animal ethics committee approval was not applicable to this work.

## Supplementary Information

Supplementary Information.

Supplementary Tables.

## Data Availability

All data generated or analysed during this study are included in the article and its Supplementary Information files. R-scripts will be published on GitHub (repository: porpoise-reproduction).
