# Supplementary material for: Nutritional status and prey energy density govern reproductive success in a small cetacean
Source: Sci Rep. 2021 Nov 2;11:19201. doi: 10.1038/s41598-021-98629-x (PMC8560860; doi:10.1038/s41598-021-98629-x)
Supplement: Supplementary file 1 — Supplementary Information. [file 41598_2021_98629_MOESM1_ESM.docx]

**Supplementary Figures**


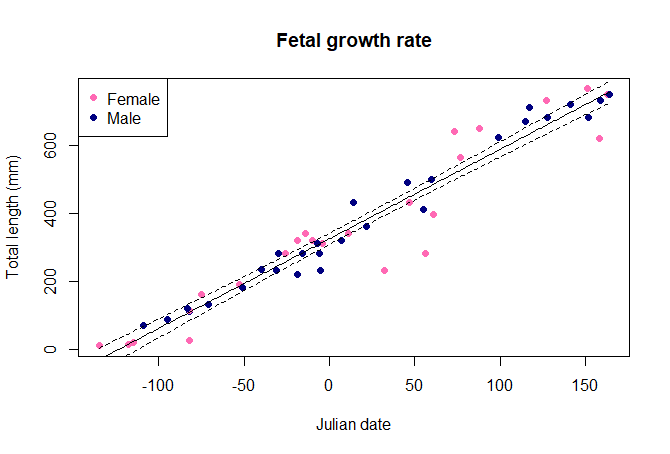
***Supplementary Figure 1.*** *Foetal growth by day (coloured by sex with pink = females, blue = males) with linear regression line. Slope of the linear regression was 2.63, indicating growth of 2.63 mm per day. The R^2^ was 0.94. Black dotted lines representing 95% CI.*

***
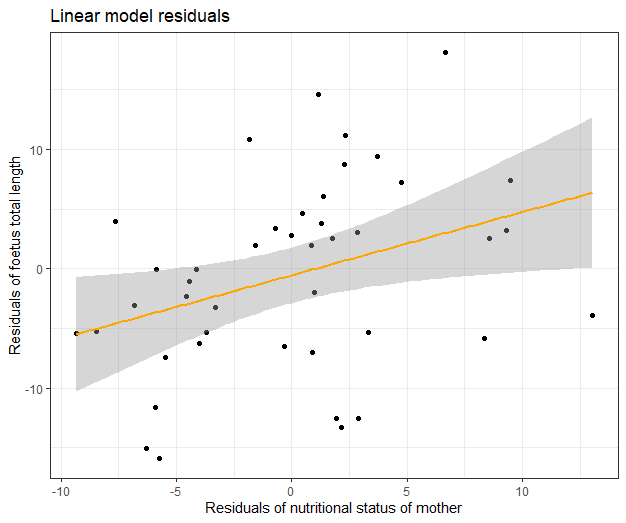
***

***Supplementary Figure 2.*** *Residuals of linear model of foetus size (total length) as a function of nutritional status of the mother reveals a positive relation between parameters. Black dotted lines representing 95% CI.*


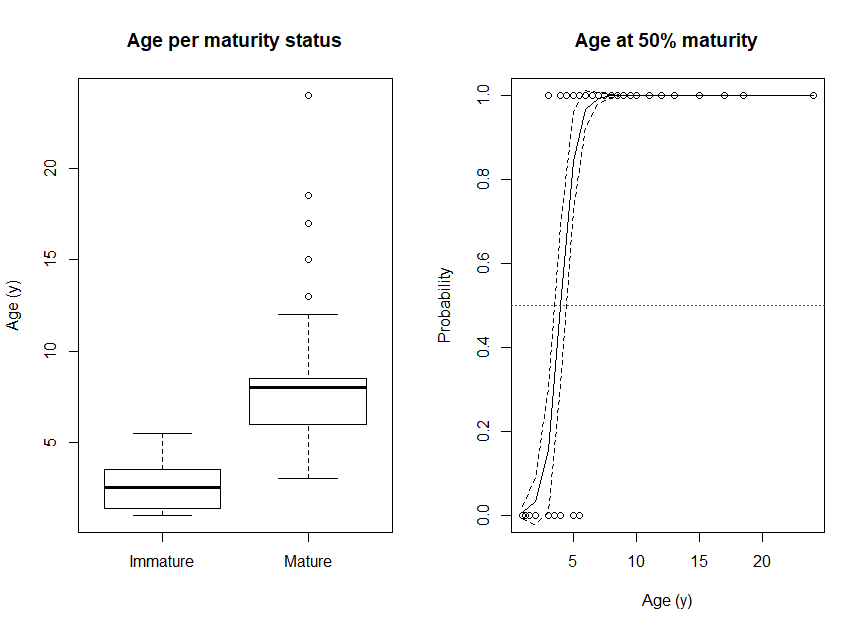


***Supplementary Figure 3.*** *Boxplot of age (n=154) per maturity status (left) and probability of age at sexual maturity (right) with fitted regression lines (black dotted lines representing 95% CI) and red dotted line at 0.5 probability.*

***
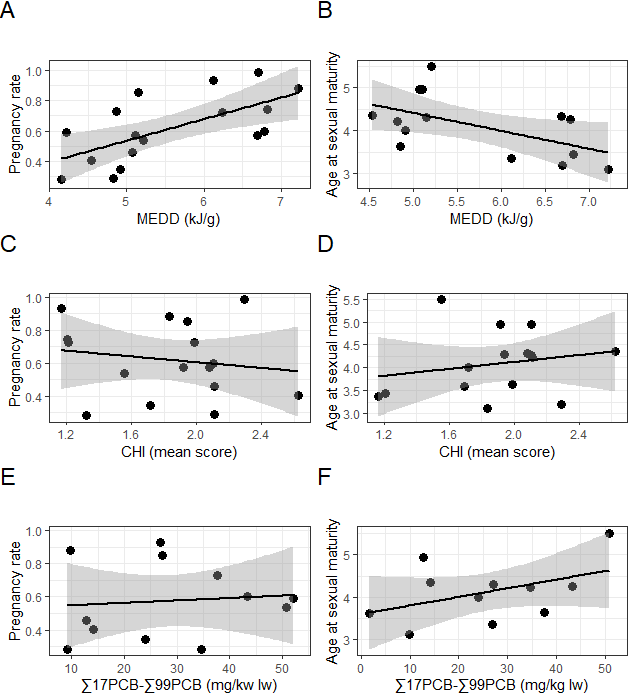
***

***Supplementary Figure 4.*** *Harbour porpoise pregnancy rate (****STab. 4****) and age at sexual maturity (****STab. 5****) in relation to environmental conditions:* ***(A, B)*** *the mean energy density of the diet (MEDD) (****STab. 6****),* ***(C,D)*** *Cumulative human impact (CHI) mean scores (****STab. 11****),* ***(E,F)*** *Chemical pollution by PCBs, restricted to studies reporting ≥∑17PCB-≤∑99PCB (****STab. 13****).* *A regression line is fitted to all graphs, with the grey shaded areas reflecting the 95% CI.*

*
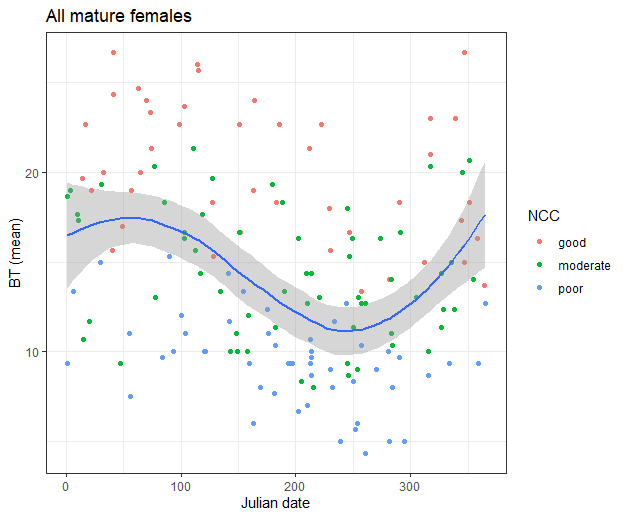
*

***Supplementary Figure 5.*** *Mean blubber thickness of mature females over the year (Julian date) shows the sinus-shaped, natural variance among all individuals. Datapoint are coloured by nutritional condition code (NCC), which are assessed during necropsies based on the animals shape, their fat reserves and musculature (see method section), with red = good, green = moderate, and blue = poor. A smooth regression line is fitted, with the grey shaded areas reflecting the 95% CI.*


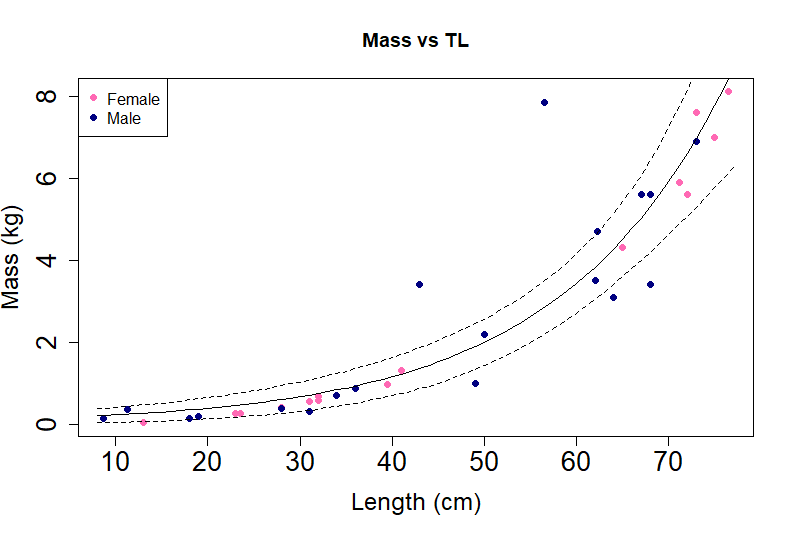
 ***Supplementary Figure 6.*** *Foetus mass versus total length (coloured by sex with pink = females, blue = males) with fitted regression line. R^2^ for foetus length as a function of mass is 0.8.* *Black dotted lines representing 95% CI.*

***Supplementary Tables***

***Supplementary Table 1****. Overview of conducted analyses and developed models in this study. Numbers in column with heading ‘Model’ corresponds to numbering used in main text.*

| **Model** | **Aim** | **Sample population** | **Procedure** |
| --- | --- | --- | --- |
| 1 | Establish a proxy of nutritional status using blubber thickness | Mature females from Dutch waters | Residuals of GAM of mean blubber thickness ~ Julian date |
| 2 | Identify the best measure for foetus size | All singular foetuses from Dutch waters | Linear model for foetus length and weight |
| 3 | Determine if foetus size could be explained by covariates: total length, nutritional status using corBT, health status of the mother, and day within gestation period | All singular foetuses from Dutch waters | GLM with gaussian distribution |
| 4 | Determine if foetus size could be explained by covariates: total length, nutritional status using NCC, health status of the mother, and day within gestation period | All singular foetuses from Dutch waters | GLM with gaussian distribution |
| 5 | Determine if pregnancy could be explained by covariates: age, year, month, nutritional status using corBT and health status of the mother | Mature females from Dutch waters | GLM with binomial error distribution and logit link |
| 6 | Determine if pregnancy could be explained by covariates: age, year, month, nutritional status using NCC and health status of the mother | Mature females from Dutch waters | GLM with binomial error distribution and logit link |
| 7 | Determine Age at Sexual Maturity | Mature and immature females from Dutch waters with an established age | GLM with binomial error distribution and logit link |
| 8 | Determine which environmental conditions best explain pregnancy rates globally | Literature; incl. Dutch waters | GLMTMB with binomial error distribution and logit link, with a random area effect |
| 9 | Determine which environmental conditions best explain age at sexual maturity globally | Literature; incl. Dutch waters | GLM with gaussian distribution, weighed by sample size |

***Supplementary Table 2****. Overview of life history and environmental condition studies; their associated time frames and references to the literature studies.*

| **Life history** | | | **Diet** | | | **Cumulative human impact** | | | **Polychlorinated biphenyls** | | |
| --- | --- | --- | --- | --- | --- | --- | --- | --- | --- | --- | --- |
| ***Location*** | ***Period*** | ***Ref*** | ***Location*** | ***Period*** | ***Ref*** | ***Location*** | ***Period*** | ***Ref*** | ***Location*** | ***Period*** | ***Ref*** |
| Bay of Fundy | 1985-1988 | ^1^ | Bay of Fundy | 1985-1987 | ^2^ | Bay of Fundy | 2008 | ^3^ | Bay of Fundy | 1971-1977 | ^4,5^ |
| Celtic & Irish waters | 1990-2013 | ^6^ | Irish waters | 1993-2011 | ^7^ | Celtic Sea, Irish Sea and St. George's Channel | 2008 | ^3^ | Celtic & Irish waters | 1990-2005 | ^5,8,9^ |
| Danish waters | 1985-1991 | ^10^ | Danish waters | 1985-1992 | ^11^ | Danish waters | 2008 | ^3^ | Danish waters | 1988-1991 | ^5,12,13^ |
| Danish waters | 1980-1981 | ^10^ recalc. in ^14^ | NA | NA | NA | NA | NA | NA | NA | NA | NA |
| Danish Little Belt | 1941-1943 | ^10^ recalc.in ^14^ | NA | NA | NA | NA | NA | NA | NA | NA | NA |
| Dutch waters - new | 2006-2019 | This study | Dutch waters new | 2006-2019 | This study | Dutch part of the North Sea/Dutch waters | 2008 | ^3^ | Dutch waters - new | 2000-2017 | ^5,15–17^ |
| Dutch waters - old | 1990-1995 | ^18^ | Dutch waters old | 1986-2003 | ^11^ | NA | NA | NA | Dutch waters - old | 1992-1998 | ^5,15,19^ |
| Eastern Newfoundland | 1990-1991 | ^20^ | Gulf of St. Lawrence | 1989 | ^21^ | Labrador - Newfoundland | 2008 | ^3^ | Newfoundland and Gulf of St. Lawrence | 1991 | ^5,22^ |
| English North Sea | 1990-2013 | ^6^ | English North Sea | 1989-1994 | ^23^ | English North Sea and Welsh waters | 2008 | ^3^ | English and Welsh waters | 1990-2012 | ^5,8,9,24^ |
| German Baltic Sea | 1990-2016 | ^25^ | German Baltic Sea | 1994-2006 | ^26^ | Baltic Sea | 2008 | ^3^ | Baltic Sea | 1988-2000 | ^5,27,28^ |
| German North Sea | 1990-2016 | ^25^ | German North Sea | 1994-2006 | ^26^ | German North Sea | 2008 | ^3^ | German North Sea | 1999-2000 | ITAW database |
| Gulf of Maine & Bay of Fundy | 1989-1993 | ^29^ | Gulf of Maine | 1989-1994 | ^30^ | Gulf of Maine | 2008 | ^3^ | Gulf of Maine & Bay of Fundy | 1991 | ^5,22,31^ |
| Icelandic waters | 1991-1997 | ^32^ | Icelandic waters | 1991-1997 | ^33^ | Icelandic waters | 2008 | ^3^ | Icelandic waters | 1991-1996 | ^5,34^ |
| Kattegat and Skagerrak Seas | 1988-1991 | ^14,35^ | Kattegat and Skagerrak | 1989-1996 | ^36^ | Kattegat and Skagerrak Seas | 2008 | ^3^ | Kattegat and Skagerrak | 1978-1990 | ^5,28^ |
| Massachusetts | 1975-1988 | ^37^ | Southern New England | 1994–2017 | ^38^ | Massachusetts bay and Cape Cod bay | 2008 | ^3^ | NA | NA | NA |
| Norwegian waters | 2016-2017 | ^39^ | Norwegian waters | 2016-2017 | ^40^ | Norwegian Sea | 2008 | ^3^ | Norwegian waters | 1988-1996 | ^5,13,28,34^ |
| NW Iberian Peninsula | 1990-2010 | ^41^ | NW Iberian Peninsula | 1990-2010 | ^41^ | Bay of Biscay, Portuguese EEZ (excl. Azores and Madeira) and Spanish part of North Atlantic Ocean | 2008 | ^3^ | NW Iberian Peninsula | 2004-2008 | ^42^ |
| Salish Sea | 1980-2015 | ^43^ | Salish Sea | 1991-2010 | ^44^ | The Coastal Waters of Southeast Alaska and British Columbia | 2008 | ^3^ | Salish Sea | 1985-1988 | ^5,45,46^ |
| Scottish waters | 1992-2005 | ^47^ | Scottish waters | 1992-2003 | ^48^ | Inner Seas off the West Coast of Scotland, Scottish North Sea, UK part of North Atlantic Ocean | 2008 | ^3^ | Scottish waters | 1989-2012 | ^5,8,9,24,49^ |
| West Greenland | 1988–1995 | ^50^ | NA | NA | NA | Davis Strait | 2008 | ^3^ | West Greenland | 1995 | ^51^ |

***Supplementary Table 3:*** *Results from literature review on global pregnancy rates (PR). Study area= as defined in the method section of the studies. Sample source = the origin of the female harbour porpoises included in the study being either strandings incl. trauma cases. directly retrieved from fisheries or a combination of the two. Time frame = is the time frame of the specimen collection gained from the methods of the studies. Npreg = the number of pregnant females in the study. Ntotal = the total number of mature females in the study. PR = Npreg / Ntotal. Conception = if and what period was excluded from the study. NA = not available. The table is organized by highest to lowest PR and colour shadings are corresponding with highest PR = greenest to lowest PR = reddest.*

| **Study area** | **Sample source** | **Timeframe** | **Npreg** | **Ntotal** | **PR** | **Conception period excluded?** | **Reference** |
| --- | --- | --- | --- | --- | --- | --- | --- |
| Icelandic waters | Directly retrieved from fisheries | 1991-1997 | 73 | 74 | 0.986 | Yes, all were bycaught in Sept-June | ^32^ |
| Gulf of Maine | Directly retrieved from fisheries | 1989-1993 | 13 | 14 | 0.929 | Yes, excl. May-Aug | ^29^ |
| Eastern-Newfoundland | Directly retrieved from fisheries | 1990-1991 | 15 | 17 | 0.882 | Yes, all sampled in June (1990) or May (1991) | ^20^ |
| Northern Norway | Directly retrieved from fisheries | 2016-2017 | 17 | 20 | 0.850 | Yes, all bycaught in autumn 2016 and spring 2017 | ^14,52^ |
| Danish Little Belt - old | Directly retrieved from fisheries | 1941-1943 | NA | NA | 0.840 | Not reported | ^10,14^ and Pers. Comm. Carl Kinze |
| Danish waters - mid | Directly retrieved from fisheries | 1980-1981 | NA | NA | 0.790 | Not reported | ^14,53^ and Pers. Comm Carl Kinze |
| Bay of Fundy | Directly retrieved from fisheries | 1985-1988 | 26 | 35 | 0.743 | Yes, > 5 August (animals were bycaught July - Sept) | ^1^ |
| Danish waters - new(est) | Directly retrieved from fisheries | 1985-1991 | 24 | 33 | 0.727 | Yes, after September | ^10^ |
| Massachusetts | Strandings, incl. trauma cases | 1975-1988 | 13 | 18 | 0.722 | No, incl. two recently pregnant lactating females | ^37^ |
| Celtic and Irish Seas | Strandings, incl. trauma cases | 1990-2013 | 36 | 60 | 0.600 | Yes, excl. May-September | ^6^ |
| Dutch waters - old | Strandings, incl. trauma cases | 1990-1995 | 15 | 26 | 0.577 | Yes, excl. June-September | ^18^ |
| German North Sea | Strandings, incl. trauma cases, and directly retrieved from fisheries | 1990-2016 | 28 | 49 | 0.571 | No, all based on CL | ^25^ |
| Kattegat/Skagerrak Seas | Strandings, incl. trauma cases, and directly retrieved from fisheries | 1988-1991 | 20 | 35 | 0.571 | No, all based on CL | ^35^ |
| NW Iberian Peninsula | Strandings, incl. trauma cases, and directly retrieved from fisheries | 1990-2010 | 7 | 13 | 0.538 | Yes, excl. June-August | ^41^ |
| German Baltic Sea | Strandings, incl. trauma cases, and directly retrieved from fisheries | 1990-2016 | 11 | 24 | 0.458 | No, all based on CL | ^25^ |
| Scottish waters | Strandings, incl. trauma cases | 1992-2005 | 17 | 42 | 0.405 | Yes, excl. 26 May to 14 September | ^47^ |
| Dutch waters - this study | Strandings, incl. trauma cases, and directly retrieved from fisheries | 2006-2019 | 46 | 161 | 0.345 | Yes, excl. May-August | This study |
| English North Sea and Welsh waters | Strandings, incl. trauma cases | 1990-2013 | 12 | 42 | 0.286 | Yes, excl. May-September | ^6^ |
| Salish Sea | Strandings, incl. trauma cases | 1980-2015 | 15 | 53 | 0.283 | Yes, excl. 1 Aug - 31 Dec | ^43^ |

***Supplementary Table 4****. Results from literature review on global age at sexual maturity (ASM). Study area= as defined in the method section of the studies. Sample source = the origin of the female harbour porpoises included in the study, being either strandings incl. trauma cases, directly retrieved from fisheries or a combination of the two. Time frame = is the time frame of the specimen collection gained from the methods of the studies. ASM = gained from the literature. Method = how the ASM was calculated. Error = given standard error (ED) or 95% confidence intervals (CI). NA=not reported. The table is organized by lowest to highest ASM.*

| **Country/area** | **Sample source** | **Timeframe** | **Ntotal** | **ASM** | **Method** | **Error** | **Reference** |
| --- | --- | --- | --- | --- | --- | --- | --- |
| Eastern-Newfoundland | Directly retrieved from fisheries | 1990-1991 | 32 | 3.1 | Age-specific ovulation rates, as ^54^ | SE=0.07 | ^20^ |
| Icelandic waters | Directly retrieved from fisheries | 1991-1997 | 354 | 3.2 | 50% maturity, as e.g. ^55^ | 2.1 to 4.4 | ^32^ |
| Gulf of Maine | Directly retrieved from fisheries | 1989-1993 | 37 | 3.36 | Sum of Fraction Immature algorithm, as ^56^ | SD=0.13 | ^29^ |
| Bay of Fundy | Strandings, incl. trauma cases, and directly retrieved from fisheries | 1985-1988 | 108 | 3.44 | Age-specific ovulation rates, as ^54^ | SE=0.182. 95%CI=3.46-4.08 | ^1^ |
| Denmark waters - old | Directly retrieved from fisheries | 1980-1981 | 78 | 3.5 | Not specified | NA | ^53^ |
| West Greenland | Directly retrieved from fisheries | 1988–1995 | 84 | 3.6 | Age-specific ovulation rates, as ^54^ | NA | ^50^ |
| Danish waters - new(est) | Strandings, incl. trauma cases, and directly retrieved from fisheries | 1985-1991 | 83 | 3.63 | Sum of Fraction Immature algorithm, as ^56^ | NA | ^10^ |
| Norwegian waters | Strandings, incl. trauma cases, and directly retrieved from fisheries | 1988-1990 | 54 | 3.9 | Not specified | ± =0.4 | ^57^ |
| Dutch waters - this sudy | Strandings, incl. trauma cases, and directly retrieved from fisheries | 2006-2019 | 154 | 4 | 50% maturity, as e.g. ^55^ | 95% CI: 3.47-4.48 | This study |
| English North Sea and Welsh waters | Strandings, incl. trauma cases, and directly retrieved from fisheries | 1990-2012 | 117 | 4.22 | 50% maturity, as e.g. ^55^ | SE=0.26 | ^58^ |
| Celtic and Irish Seas | Strandings, incl. trauma cases | 1990-2012 | 188 | 4.26 | 50% maturity, as e.g. ^55^ | SE=0.27 | ^58^ |
| Northern Norway | Directly retrieved from fisheries | 2016-2017 | 48 | 4.3 | ^54^/Richards’ curves as ^59^ | SE=0.6 | ^14^ |
| Swedish Kattegat and Skagerrak Seas | Strandings, incl. trauma cases, and directly retrieved from fisheries | 1988-1991 | 202 | 4.32 | Age-specific ovulation rates, as ^54^ | SD=0.29. 95%CI: 3.76-4.87 | ^35^ |
| Scottish waters | Strandings, incl. trauma cases, and directly retrieved from fisheries | 1992-2005 | 144 | 4.35 | 50% maturity, as e.g. ^55^ | 95%CI=3.9-4.71 | ^47^ |
| German North Sea | Strandings and bycatch | 1990-2016 | 69 | 4.95 | 50% maturity, as e.g. ^55^ | ± =0.6 | ^25^ |
| German Baltic Sea | Strandings, incl. trauma cases, and directly retrieved from fisheries | 1990-2016 | 42 | 4.95 | 50% maturity, as e.g. ^55^ | ± =0.6 | ^25^ |
| NW Iberian Peninsula | Strandings, incl. trauma cases, and directly retrieved from fisheries | 1990-2009 | 48 | 5.5 | 50% maturity, as e.g. ^55^ | NA | ^41^ |

***Supplementary Table 5****. Summary table of the results from literature review on mean energetic density of porpoise diet (MEDD). Study area= as defined in the method section of the studies. Time frame = is the time frame of the specimen collection gained from the methods of the studies. MEDD = calculated (see methods). Most abundant prey (%M) = the most abundant species in the diet and the percentage of the mass of that species in the total diet in parentheses. The table is organized by highest to lowest MEDD and colour shadings are corresponding, with highest MEDD = greenest to lowest MEDD = reddest.*

| **Study area** | **MEDD** | **Most abundant prey (%M)** | **Ntotal** | **Nnon-empty** | **Reference** |
| --- | --- | --- | --- | --- | --- |
| Gulf of St. Lawrence | 7.22 | *Clupea harengus* (43) | 138 | 111 | ^21^ |
| Bay of Fundy | 6.82 | *Clupea harengus* (64) | 160 | 127 | ^2^ |
| Icelandic waters | 6.7 | *Mallotus villosus* (78) | 1047 | 1012 | ^33^ |
| Kattegat and Skagerrak | 6.69 | *Clupea harengus* (50) | 112 | 112 | ^36^ |
| Southern New England | 6.24 | *Urophycis chuss* (17) | 50 | 46 | ^38^ |
| Gulf of Maine | 6.12 | *Merluccius bilinearis* (43) | 95 | 95 | ^30^ |
| Irish waters | 5.78 | *Clupea harengus* (39) | 66 | 66 | ^7^ |
| NW Iberian Peninsula | 5.21 | *Trisopterus sp* (32) | 56 | 56 | ^41^ |
| Norwegian Sea | 5.15 | *Pollachius virens* (57) | 134 | 122 | ^40^ |
| German North Sea | 5.11 | *Gobiidae sp* (26) | 62 | 62 | ^26^ |
| German Baltic Sea | 5.07 | *Gadus morhua* (64) | 53 | 53 | ^26^ |
| Dutch waters - this study | 4.92 | *Merlangius merlangus* (41) | 1457 | 985 | This study |
| Danish waters | 4.86 | *Gadus morhua* (28) | 58 | 58 | ^11^ |
| English North Sea | 4.82 | *Gadus morhua* (21) | 17 | 17 | ^23^ |
| Scottish waters | 4.54 | *Merlangius merlangus* (52) | 188 | 188 | ^48^ |
| Dutch waters - old | 4.21 | *Merlangius merlangus* (77) | 90 | 90 | ^11^ |
| Salish Sea | 4.15 | *Malacocottus kincaidi* (80) | 36 | 31 | ^44^ |

***Supplementary Table 6.*** *Distribution of %M over different prey quality categories, with %M≥ 6 kJ·g-1 reflecting high energy density prey,* *%M4-6 kJ·g-1 reflecting moderate energy density prey, and* *% M≤ 4 kJ·g-1 reflecting low energy density prey.*

| **Location of diet study** | **MEDD** | **High energy** | **Moderate energy** | **Low energy** | **Reference** |
| --- | --- | --- | --- | --- | --- |
|  | *kJ·g-1* | *%M≥ 6 kJ·g-1* | *%M4-6 kJ·g-1* | *% M≤ 4 kJ·g-1* |  |
| Salish Sea | 4.15 | 13.43 | 0.05 | 86.52 | ^44^ |
| Dutch waters - old | 4.21 | 2.96 | 19.41 | 77.63 | ^11^ |
| Scottish waters | 4.54 | 3.71 | 44.39 | 51.90 | ^48^ |
| English North Sea | 4.82 | 10.70 | 70.33 | 18.97 | ^23^ |
| Danish waters | 4.86 | 12.10 | 74.77 | 13.13 | ^11^ |
| Dutch waters - new | 4.92 | 17.37 | 40.53 | 42.10 | This study |
| German Baltic Sea | 5.07 | 17.88 | 78.97 | 3.14 | ^26^ |
| German North Sea | 5.11 | 6.58 | 88.82 | 4.59 | ^26^ |
| Norwegian waters | 5.15 | 15.11 | 83.89 | 1.00 | ^40^ |
| NW Iberian Peninsula | 5.21 | 28.48 | 61.57 | 9.95 | ^41^ |
| Irish waters | 5.78 | 42.32 | 48.93 | 8.76 | ^7^ |
| Gulf of Maine | 6.12 | 28.69 | 71.31 | 0.00 | ^30^ |
| Southern New England | 6.24 | 29.54 | 68.47 | 1.99 | ^38^ |
| Kattegat and Skagerrak | 6.69 | 70.24 | 24.91 | 4.85 | ^36^ |
| Icelandic waters | 6.70 | 78.02 | 17.83 | 4.15 | ^33^ |
| Bay of Fundy | 6.82 | 65.60 | 34.40 | 0.00 | ^2^ |
| Gulf of St. Lawrence | 7.22 | 87.85 | 12.15 | 0.00 | ^21^ |

***Supplementary Table 7.*** *Caloric values of prey species (Family and Scientific names provided) with the energy density (ED) in kJ/g wet weight.* See Excel datasheet ‘ST7_ED’. ***Supplementary Table 8.*** *Mean energy density of the diet (MEDD) calculation per study area where mass was reported in grams (g).* See Excel datasheet ‘ST8_MEDDlog_g’. References: ^60,61,70–79,62,80–89,63,90–97,64–69^

***Supplementary Table 9.*** *Mean energy density of the diet (MEDD) calculation per study area where mass was reported as percentage (%M).*

| **Location** | **Prey species** | **Reconstructed mass (%M)** | **ED*** | **Product** | **Reference** | **Remarks** |
| --- | --- | --- | --- | --- | --- | --- |
|  |  |  | *(kJ/g)* |  | *Reconstructed mass* |  |
| Scottish waters | *Ammodytidae sp* | 24.8 | 5.5 | 135.5 | ^48^ | Value for mean *Ammodytidae* |
| Scottish waters | *Trachurus trachurus* | 0.01 | 6.3 | 0.1 | ^48^ |  |
| Scottish waters | *Clupeidae sp* | 0.4 | 8.5 | 3.7 | ^48^ |  |
| Scottish waters | *Clupea harengus* | 1.4 | 7.8 | 11.1 | ^48^ |  |
| Scottish waters | *Sprattus sprattus* | 0.4 | 8.1 | 2.8 | ^48^ |  |
| Scottish waters | *Gadidae sp* | 4.3 | 4.4 | 18.9 | ^48^ |  |
| Scottish waters | *Trisopterus sp* | 3.7 | 4.7 | 17.3 | ^48^ |  |
| Scottish waters | *Gadus morhua* | 0.6 | 4.5 | 2.7 | ^48^ |  |
| Scottish waters | *Melanogrammus aeglefinus* | 1.4 | 4.0 | 5.5 | ^48^ |  |
| Scottish waters | *Merlangius merlangus* | 51.7 | 3.9 | 201.4 | ^48^ |  |
| Scottish waters | *Micromesistius poutassou* | 0.2 | 4.4 | 1.0 | ^48^ |  |
| Scottish waters | *Pollachius sp + M. aeglefinus* | 6.1 | 4.3 | 26.7 | ^48^ | Value for *P. pollachius, P. virens, M. aeglefinus* |
| Scottish waters | *Gobiidae sp* | 0.03 | 5.0 | 0.1 | ^48^ |  |
| Scottish waters | *Rhinonemus cimbrius* | 0.02 | 4.5 | 0.1 | ^48^ | Value for *Enchelyopus cimbrius* |
| Scottish waters | *Scomber scombrus* | 1.5 | 7.7 | 11.5 | ^48^ |  |
| Scottish waters | *Alloteuthis sp* | 0.2 | 3.9 | 0.6 | ^48^ |  |
| Scottish waters | *Sepiola sp.* | 3.3 | 4.8 | 15.7 | ^48^ |  |
| Scottish waters | *Cephalopds* | 0.01 | 4.4 | 0.04 | ^48^ |  |
|  |  | 100.0 |  | 454.4 |  |  |
|  |  | **MEDD =** | **4.544** |  |  |  |
|  | | | | | | |
| German Baltic Sea | *Gadus morhua* | 63.3 | 4.5 | 285.2 | ^26^ |  |
| German Baltic Sea | *Gobiidae sp* | 5.6 | 5.0 | 27.7 | ^26^ |  |
| German Baltic Sea | *Clupea harengus* | 13.3 | 7.8 | 103.5 | ^26^ |  |
| German Baltic Sea | *Solea solea* | 3.8 | 5.0 | 19.1 | ^26^ |  |
| German Baltic Sea | *Merlangius merlangus* | 3.1 | 3.9 | 12.1 | ^26^ |  |
| German Baltic Sea | *Zoarces vivaparus* | 5.3 | 4.1 | 21.4 | ^26^ |  |
| German Baltic Sea | *Sprattus sprattus* | 2.1 | 8.1 | 17.2 | ^26^ |  |
| German Baltic Sea | *Trachurus trachurus* | 2.3 | 6.3 | 14.2 | ^26^ |  |
|  |  | 98.7 |  | 500.2 | ^26^ |  |
|  |  | **MEDD =** | **5.068** |  | ^26^ |  |
|  | | | | | | |
| German North Sea | *Gadus morhua* | 21.0 | 4.5 | 94.6 | ^26^ |  |
| German North Sea | *Gobiidae sp* | 25.4 | 5.0 | 125.7 | ^26^ |  |
| German North Sea | *Ammodytes sp* | 20.6 | 5.6 | 116.1 | ^26^ |  |
| German North Sea | *Clupea harengus* | 4.2 | 7.8 | 32.9 | ^26^ |  |
| German North Sea | *Solea solea* | 12.9 | 5.0 | 64.6 | ^26^ |  |
| German North Sea | *Merlangius merlangus* | 4.5 | 3.9 | 17.4 | ^26^ |  |
| German North Sea | *Limanda limanda* | 4.5 | 4.6 | 20.4 | ^26^ |  |
| German North Sea | *Sprattus sprattus* | 1.4 | 8.1 | 11.7 | ^26^ |  |
| German North Sea | *Trachurus trachurus* | 0.8 | 6.3 | 4.7 | ^26^ |  |
| German North Sea | *Hyperoplus lanceolatus* | 2.2 | 4.8 | 10.7 | ^26^ |  |
|  |  | 97.53 |  | 498.8 |  |  |
|  |  | **MEDD =** | **5.114** |  |  |  |
|  | | | | | | |
| Danish waters | *Anguilla anguilla* | 0.6 | 5.7 | 3.4 | ^11^ |  |
| Danish waters | *Clupea harengus* | 11.1 | 7.8 | 86.4 | ^11^ |  |
| Danish waters | *Gadus morhua* | 27.5 | 4.5 | 123.9 | ^11^ |  |
| Danish waters | *Melanogrammus aeglefinus* | 0.5 | 4.0 | 2.0 | ^11^ | Value for *P. pollachius, P. virens, M. aeglefinus* |
| Danish waters | *Pollachius sp, + M. aeglefinus* | 0.8 | 4.3 | 3.5 | ^11^ |  |
| Danish waters | *Pollachius sp* | 1.1 | 4.4 | 5.0 | ^11^ |  |
| Danish waters | *Merlangius merlangus* | 12.1 | 3.9 | 47.1 | ^11^ |  |
| Danish waters | *Trisopterus sp* | 0.1 | 4.7 | 0.5 | ^11^ |  |
| Danish waters | *Labridae sp* | 0.6 | 4.7 | 2.8 | ^11^ | Value for *Labrus bergylta* |
| Danish waters | *Zoarces vivaparus* | 25.7 | 4.1 | 104.8 | ^11^ |  |
| Danish waters | *Ammodytes sp* | 11.5 | 5.6 | 64.7 | ^11^ |  |
| Danish waters | *Gobiidae sp* | 3.9 | 5.0 | 19.3 | ^11^ |  |
| Danish waters | *Scomber scombrus* | 0.6 | 7.7 | 4.6 | ^11^ |  |
| Danish waters | *Alloteuthis subulata* | 0.6 | 3.9 | 2.3 | ^11^ | Value for *Alloteuthis sp* |
|  |  | 96.7 |  | 470.3 |  |  |
|  |  | **MEDD =** | **4.863** |  |  |  |
|  | | | | | | |
| Dutch waters old | *Clupea harengus* | 2.2 | 7.8 | 17.1 | ^11^ |  |
| Dutch waters old | *Sprattus sprattus* | 0.2 | 8.1 | 1.6 | ^11^ |  |
| Dutch waters old | *Gadus morhua* | 3.8 | 4.5 | 17.1 | ^11^ |  |
| Dutch waters old | *Pollachius sp, + M. aeglefinus* | 0.1 | 4.3 | 0.4 | ^11^ | Value for *P. pollachius, P. virens, M. aeglefinus* |
| Dutch waters old | *Merlangius merlangus* | 75.7 | 3.9 | 294.2 | ^11^ |  |
| Dutch waters old | *Trisopterus sp* | 1.3 | 4.7 | 6.1 | ^11^ |  |
| Dutch waters old | *Mugilidae sp* | 0.1 | 6.5 | 0.7 | ^11^ | Value for *Liza ramada* |
| Dutch waters old | *Ammodytes sp* | 4.2 | 5.6 | 23.6 | ^11^ |  |
| Dutch waters old | *Gobiidae sp* | 7.5 | 5.0 | 37.1 | ^11^ |  |
| Dutch waters old | *Trachurus sp* | 0.4 | 6.3 | 2.5 | ^11^ | Value for *Trachurus trachurus* |
| Dutch waters old | *Sepiolidae sp* | 0.2 | 4.8 | 1.0 | ^11^ | Value for *Sepiola sp* |
| Dutch waters old | *Sepiola atlantica* | 0.1 | 4.8 | 0.5 | ^11^ | Value for *Sepiola sp* |
| Dutch waters old | *Alloteuthis subulate* | 0.3 | 3.9 | 1.2 | ^11^ | Value for *Alloteuthis sp* |
| Dutch waters old | *Loligo forbesi* | 1.6 | 4.8 | 7.7 | ^11^ |  |
| Dutch waters old | *Cephalopds* | 0.2 | 4.4 | 0.9 | ^11^ |  |
|  |  | 97.9 |  | 412.2 |  |  |
|  |  | **MEDD =** | **4.21** |  |  |  |
|  | | | | | | |
| Norwegian waters | *Ammodytes sp* | 0.3 | 5.6 | 1.5 | ^40^ |  |
| Norwegian waters | *Clupea harengus* | 5.1 | 7.8 | 39.4 | ^40^ |  |
| Norwegian waters | *Gadiculus argenteus thori* | 1.8 | 5.0 | 9.0 | ^40^ |  |
| Norwegian waters | *Gadus morhua* | 1.8 | 4.5 | 8.2 | ^40^ |  |
| Norwegian waters | *Melanogrammus aeglefinus* | 0.8 | 4.0 | 3.3 | ^40^ |  |
| Norwegian waters | *Merlangius merlangus* | 0.8 | 3.9 | 3.2 | ^40^ |  |
| Norwegian waters | *Micromesistius poutassou* | 10.5 | 4.4 | 46.5 | ^40^ |  |
| Norwegian waters | *Pollachius virens* | 57.5 | 4.8 | 277.5 | ^40^ | *Trisopterus miniutus, Trisopterus esmarkii* |
| Norwegian waters | *Trisopterus sp* | 8.4 | 4.7 | 39.4 | ^40^ |  |
| Norwegian waters | *Gadidae sp* | 1.8 | 4.4 | 7.9 | ^40^ |  |
| Norwegian waters | *Leptoclinus maculatus* | 0.2 | 5.4 | 1.0 | ^40^ |  |
| Norwegian waters | *Lumpenus lampretaeformis* | 0.01 | 5.2 | 0.1 | ^40^ | Value for *Lumpenus sp* |
| Norwegian waters | *Liparis sp* | 0.02 | 3.5 | 0.1 | ^40^ |  |
| Norwegian waters | *Merluccius merluccius* | 0.1 | 3.9 | 0.4 | ^40^ |  |
| Norwegian waters | *Mallotus villosus* | 6.4 | 7.1 | 45.3 | ^40^ |  |
| Norwegian waters | *Sebastes sp* | 0.02 | 4.6 | 0.1 | ^40^ |  |
| Norwegian waters | *Scomber scombrus* | 3.7 | 7.7 | 28.1 | ^40^ |  |
| Norwegian waters | *Decapods* | 0.03 | 4.2 | 0.1 | ^40^ |  |
| Norwegian waters | *Euphausiidae sp* | 0.1 | 3.8 | 0.2 | ^40^ | Value for *Meganyctiphanes norvegica* |
| Norwegian waters | *Cephalopods* | 0.8 | 4.4 | 3.5 | ^40^ | Value for Cephalopods |
|  |  | 100 |  | 514.9 |  |  |
|  |  | **MEDD =** | **5.149** |  |  |  |
|  | | | | | | |
| Icelandic waters | *Mallotus villosus* | 76.6 | 7.1 | 543.1 | ^33^ |  |
| Icelandic waters | *Ammodytes sp.* | 13.9 | 5.6 | 78.2 | ^33^ |  |
| Icelandic waters | *Merlangius merlangus* | 4.1 | 3.9 | 15.9 | ^33^ |  |
| Icelandic waters | *Sebastes marinus* | 3.6 | 5.5 | 19.8 | ^33^ |  |
|  |  | 98.1 |  | 657.0 |  |  |
|  |  | **MEDD =** | **6.697** |  |  |  |
|  | | | | | | |
| Bay of Fundy | *Clupea harengus* | 63.8 | 7.8 | 496.6 | ^2^ |  |
| Bay of Fundy | *Merluccius bilinearis* | 18.6 | 5.3 | 98.4 | ^2^ |  |
| Bay of Fundy | *Gadus morhua* | 13.7 | 4.5 | 61.7 | ^2^ |  |
| Bay of Fundy | *Urophysis sp* | 1.8 | 5.9 | 10.6 | ^2^ | Value for *Urophycis tenuis* |
| Bay of Fundy | *Scomber scombrus* | 1.5 | 7.7 | 11.5 | ^2^ |  |
| Bay of Fundy | *Illex illecebrosus* | 0.3 | 6.5 | 2.0 | ^2^ |  |
| Bay of Fundy | *Loligo pealei* | 0.1 | 5.6 | 0.6 | ^2^ |  |
| Bay of Fundy | *Bathypolypus arcticus* | 0.2 | 4.9 | 1.0 | ^2^ | Value for *Bathypolypus signatus* |
|  |  | 100.0 |  | 682.4 |  |  |
|  |  | **MEDD =** | **6.824** |  |  |  |
|  | | | | | | |
| Gulf of St. Lawrence | *Mallotus villosus* | 37.6 | 7.1 | 266.7 | ^21^ |  |
| Gulf of St. Lawrence | *Clupea harengus* | 43.0 | 7.8 | 334.7 | ^21^ |  |
| Gulf of St. Lawrence | *Sebastes marinus* | 10.3 | 5.5 | 56.9 | ^21^ |  |
| Gulf of St. Lawrence | *Scomber scombrus* | 7.0 | 7.7 | 53.6 | ^21^ |  |
| Gulf of St. Lawrence | *Gadus morhua* | 1.4 | 4.5 | 6.3 | ^21^ |  |
| Gulf of St. Lawrence | *Illex illecebrosus* | 0.6 | 6.5 | 3.9 | ^21^ |  |
| Gulf of St. Lawrence | *Ammodytidae sp* | 0.5 | 5.5 | 2.7 | ^21^ |  |
|  |  | 100.4 |  | 724.9 |  |  |
|  |  | **MEDD =** | **7.22** |  |  |  |
| *The origin and references of the energy values can be found in ST8_ED | | | | | | |

***Supplementary Table 10.*** *Cumulative human impact (CHI) mean scores, median scores, min and maximum scores of the seventeen study areas.*

| **Study_area** | **CI_mean** | **CI_median** | **CI_min** | **CI_max** | **Reference** |
| --- | --- | --- | --- | --- | --- |
| Celtic & Irish waters | 2.106 | 2.101 | 0.366 | 6.070 | ^3,98^ |
| Kattegat Skagerrak Seas | 2.080 | 2.109 | 0.823 | 7.629 | ^3,98^ |
| German North Sea | 1.916 | 1.973 | 0.924 | 8.160 | ^3,98^ |
| Danish waters | 1.987 | 2.064 | 0.753 | 5.844 | ^3,98^ |
| Dutch waters | 1.718 | 1.938 | 0.658 | 8.391 | ^3,98^ |
| Eastern Newfoundland | 1.833 | 1.923 | 0.220 | 6.885 | ^3,98^ |
| English North Sea | 2.110 | 2.038 | 0.624 | 7.910 | ^3,98^ |
| Baltic Sea | 2.106 | 2.240 | 0.588 | 7.044 | ^3,98^ |
| Gulf of Maine | 1.168 | 1.159 | 0.185 | 4.864 | ^3,98^ |
| Bay of Fundy | 1.208 | 1.149 | 0.156 | 4.927 | ^3,98^ |
| Icelandic waters | 2.290 | 2.353 | 0.321 | 6.944 | ^3,98^ |
| Norwegian waters | 1.942 | 1.910 | 0.351 | 7.911 | ^3,98^ |
| Iberian Peninsula | 1.555 | 1.510 | 0.353 | 7.221 | ^3,98^ |
| Salish Sea | 1.323 | 1.336 | 0.251 | 6.188 | ^3,98^ |
| Scottish waters | 2.624 | 2.731 | 0.527 | 6.459 | ^3,98^ |
| West Greenland | 1.697 | 1.701 | 0.181 | 3.401 | ^3,98^ |
| Massachussets | 1.212 | 0.987 | 0.395 | 4.864 | ^3,98^ |

***Supplementary Table 11.*** *Polychlorinated biphenyls data, gained from the IWC ‘POP Contaminants Trend Explorer’ tool, hosted on the portal of the Sea Mammal Research Unit, literature and own databases (see: Source). Lipid weight of sum-PCBs are provided. See Excel datasheet ‘ST11_PCB_data’.*

***Supplementary Table 12.*** *Summary data PCB for all studies (top table, in main text referred to as PCB1) and studies restricting to ≥∑17PCB-≤∑99PCB (bottom table, in main text referred to as PCB2).*

| **ALL studies** | | | | | |
| --- | --- | --- | --- | --- | --- |
| **Location** | **Century/ies** | **PCB_sum** | **n=** | **Conc_lw** | **Reference** |
| West Greenland | 1980s-1990s | sum25 | 13 | 1.594 | ^51^ |
| German North Sea | 1990s-2000s | sum12 | 2 | 2.195 | ITAW database |
| Iceland | 1990s | sum7 | 3 | 3.028 | ^34^ |
| Eastern-Newfoundland | 1990s | sum99 | 50 | 11.141 | ^4,22^ |
| Salish Sea | 1980s | sum35; Aroclor | 9 | 13.664 | ^45,46^ |
| Scottish waters | 1990s-2010s | sum 18-25CBs; Aroclor 1254 | 127 | 14.252 | ^8,9,24,49^ |
| Northern Norway | 1980s-1900s | sum6-47 | 27 | 21.3132 | ^13,28,34^ |
| Dutch waters - new | 2000s-2010s | sum25-35 | 15 | 23.996 | ^15–17^ |
| Swedisch Kattegat and Skagerrak Seas | 1970s-1990s | sum6 | 12 | 24.250 | ^28^ |
| Gulf of Maine | 1990s | sum17-99 | 24 | 26.804 | ^22,31^ |
| Baltic Sea | 1980s-1990s | sum6-17 | 8 | 27.492 | ^27,28^ |
| Danish waters | 1980s-1990s | sum7-47 | 5 | 29.75 | ^12,13^ |
| English and Welsh waters | 1990s-2010s | sum18-25 | 102 | 34.645 | ^8,9,24^ |
| Celtic and Irish Seas | 1990s-2000s | sum25 | 18 | 43.296 | ^8,9^ |
| Iberian Peninsula | 2000s-2010s | sum32 | 1 | 50.8 | ^42^ |
| Dutch waters - old | 1990s | sum18-35 | 6 | 52.105 | ^15,19^ |
| Bay of Fundy | 1970s | Uncleard, likely Aroclor 1254 and 1260 | 62 | 82.795 | ^4^ |
| TOTAAL |  |  | 484 |  |  |
| **Restricted to ≥∑17PCB-≤∑99PCB** | | | | | |
| **Location** | **Century/ies** | **PCB_sum** | **n=** | **Conc_lw** | **Reference** |
| West Greenland | 1980s-1990s | sum25 | 13 | 1.594 | ^51^ |
| Salish Sea | 1980s | sum35 | 7 | 9.130 | ^46^ |
| Eastern-Newfoundland | 1990s | sum99 | 49 | 9.779 | ^22^ |
| Baltic Sea | 1980s-1990s | sum17 | 2 | 12.792 | ^27^ |
| Scottish waters | 1990s-2010s | sum 18-25CBs | 117 | 14.057 | ^8,9,24^ |
| Dutch waters - new | 2000s-2010s | sum25-35 | 15 | 23.996 | ^15–17^ |
| Gulf of Maine | 1990s | sum17-99 | 24 | 26.804 | ^22,31^ |
| Northern Norway | 1980s-1900s | sum47 | 16 | 27.144 | ^13^ |
| English and Welsh waters | 1990s-2010s | sum18-25 | 102 | 34.645 | ^8,9,24^ |
| Danish waters | 1990s | sum47 | 3 | 37.583 | ^13^ |
| Celtic and Irish Seas | 1990s-2000s | sum25 | 18 | 43.296 | ^8,9^ |
| Iberian Peninsula | 2000s-2010s | sum32 | 1 | 50.800 | ^42^ |
| Dutch waters - old | 1990s | sum18-35 | 6 | 52.105 | ^15,19^ |
| German North Sea | NA | NA | NA | NA | NA |
| Swedisch Kattegat and Skagerrak Seas | NA | NA | NA | NA | NA |
| Bay of Fundy | NA | NA | NA | NA | NA |
| Iceland | NA | NA | NA | NA | NA |
| TOTAAL |  |  | 373 |  |  |

***Supplementary Table 13.*** *Case information of harbour porpoises necropsied in the Netherlands, selection of cases: >115 cm total length (n=337) and used for assessment of maturity and reproductive status.*

| **Idcode** | **Ageclass** | **Age** | **Sex** | **DCC** | **Mass** | **Mass_real** | **TL** | **TL_real** | **BT_av** | **BT_real** | **NCC** | **Pregnant** | **Maturity** |
| --- | --- | --- | --- | --- | --- | --- | --- | --- | --- | --- | --- | --- | --- |
| TX064 | A | 7 | F | 2 | 52 | Almost | 144 | Yes | 26.7 | Yes | good | No | Mature |
| TX037 | A | 7.5 | F | 4 | 41 | No | 142 | Yes | 19.0 | Almost | good | Yes | Mature |
| TX004 | A | 7.5 | F | 1 | 42 | Yes | 147 | Yes | 20.0 | Yes | good | No | Mature |
| TX062 | A | 7 | F | 4 |  | No | 156 | Almost |  | No | good | NE | Mature |
| TX048 | J | 1.25 | F | 4 | 17 | Almost | 122 | Yes | 4.0 | Yes | poor | No | Immature |
| TX049 | A | 7 | F | 4 | 33 | Almost | 158 | Almost | 5.0 | Yes | moderate | No | Mature |
| TX050 | A | 7 | F | 4 |  | No | 142 | Almost |  | No | Unknown | NE | Mature |
| TX054 | A |  | F | 4 |  | No | 144 | Almost |  | No | Unknown | NE | Unknown |
| UT052 | A | 9 | F | 4 | 45 | Almost | 160 | Yes | 26.7 | Yes | good | Yes | Mature |
| UT015 | A |  | F | 2 | 38 | Yes | 144 | Yes | 10.7 | Yes | moderate | No | Mature |
| UT051 | A |  | F | 4 | 34 | Almost | 140 | Yes | 9.3 | Yes | good | NE | Unknown |
| UT035 | J |  | F | 3 |  | No | 130 | Yes | 24.3 | Yes | good | No | Immature |
| UT045 | A |  | F | 3 |  | No | 140 | Yes | 21.3 | Yes | good | No | Mature |
| UT039 | J |  | F | 2 | 23 | Yes | 122 | Yes | 9.3 | Yes | moderate | No | Immature |
| UT046 | J |  | F | 2 | 23 | Yes | 127 | Yes | 5.3 | Yes | poor | No | Immature |
| UT022 | A |  | F | 1 | 39 | Yes | 156 | Yes | 12.0 | Yes | poor | No | Mature |
| UT058 | A |  | F | 4 | 32 | Almost | 151 | Yes | 8.0 | Yes | poor | Unknown | Unknown |
| UT003 | J |  | F | 1 | 22 | Yes | 124 | Yes | 9.3 | Yes | moderate | No | Immature |
| UT033 | A | 8 | F | 4 | 35 | Almost | 161 | Yes | 9.0 | Yes | moderate | No | Mature |
| UT053 | A |  | F | 4 | 45 | Almost | 156 | Yes | 13.0 | Yes | moderate | No | Mature |
| UT048 | A |  | F | 3 | 31 | Almost | 150 | Yes | 12.7 | Yes | moderate | No | Mature |
| UT041 | A |  | F | 3 | 45 | Almost | 157 | Yes | 8.0 | Yes | poor | NE | Unknown |
| UT014 | A |  | F | 4 | 44.2 | Almost | 164 | Yes | 13.0 | Yes | moderate | No | Mature |
| UT149 | A |  | F | 3 | 39.5 | Almost | 154 | Yes | 10.0 | Yes | poor | No | Mature |
| UT126 | A |  | F | 3 | 58.5 | Almost | 156 | Yes | 19.0 | Yes | good | Yes | Mature |
| UT152 | J |  | F | 2 | 22 | Yes | 125 | Yes | 8.0 | Yes | poor | No | Immature |
| UT175 | J |  | F | 3 | 17.5 | Almost | 122 | Yes | 5.5 | Yes | poor | No | Immature |
| UT128 | J |  | F | 3 |  | No | 128 | Yes | 14.0 | Yes | moderate | No | Immature |
| UT180 | J |  | F | 3 | 17.5 | Yes | 129 | Yes | 3.7 | Yes | poor | No | Immature |
| UT173 | J |  | F | 1 | 22.5 | Yes | 127 | Yes | 8.0 | Yes | poor | No | Immature |
| UT169 | A |  | F | 2 | 48 | Yes | 165 | Yes | 15.3 | Yes | moderate | No | Mature |
| UT140 | A |  | F | 4 | 39 | No | 156 | Almost | 12.0 | Yes | moderate | NE | Unknown |
| UT151 | A |  | F | 4 | 45 | Almost | 151 | Almost | 12.0 | Yes | moderate | NE | Unknown |
| UT179 | A | 8 | F | 1 | 37 | Yes | 152 | Yes | 8.0 | Yes | poor | Yes | Mature |
| UT178 | J |  | F | 3 | 26.6 | Almost | 124 | Yes | 15.0 | Yes | moderate | No | Immature |
| UT181 | J |  | F | 1 | 40.5 | Yes | 132 | Yes | 20.0 | Yes | good | No | Immature |
| UT102 | A |  | F | 2 | 57.5 | Almost | 152 | Yes | 23.0 | Yes | good | No | Mature |
| UT108 | J |  | F | 1 | 28.5 | Yes | 139 | Yes | 11.3 | Yes | moderate | No | Immature |
| UT172 | A |  | F | 2 | 44 | Yes | 152 | Yes | 11.3 | Yes | moderate | No | Mature |
| UT176 | A |  | F | 2 | 33 | Yes | 145 | Yes | 14.3 | Yes | moderate | No | Mature |
| UT228 | J | 5.5 | F | 2 | 33.6 | Yes | 129.5 | Yes | 18.7 | Yes | good | No | Immature |
| UT243 | J | 3 | F | 2 | 30.5 | Yes | 124.5 | Yes | 23.7 | Yes | good | No | Immature |
| UT202 | A |  | F | 3 | 46 | Yes | 158 | Yes | 9.3 | Yes | poor | No | Mature |
| UT231 | J | 1 | F | 2 | 31.5 | Yes | 121 | Yes | 24.0 | Yes | good | No | Immature |
| UT236 | A | 8 | F | 3 | 53.5 | Yes | 161 | Almost | 16.7 | Yes | moderate | No | Mature |
| UT311 | A |  | F | 3 | 35.5 | Yes | 151 | Yes | 10.0 | Yes | moderate | No | Mature |
| UT318 | A | 4 | F | 3 | 37 | Almost | 146.5 | Yes | 13.3 | Yes | poor | No | Mature |
| UT261 | A | 8 | F | 3 | 34.5 | Almost | 151 | Yes | 9.3 | Yes | poor | No | Mature |
| UT271 | A | 8 | F | 4 | 43.5 | Yes | 160 | Yes | 9.3 | Yes | poor | No | Mature |
| UT302 | J |  | F | 3 | 17 | No | 122 | No | 2.7 | Almost | poor | No | Immature |
| UT255 | A |  | F | 2 | 36.5 | Yes | 157.5 | Yes | 8.7 | Yes | poor | No | Mature |
| UT284 | J | 5 | F | 3 | 37 | Yes | 137.5 | Yes | 18.7 | Yes | good | No | Immature |
| UT334 | A |  | F | 4 | 48 | Almost | 159.5 | Almost | 16.3 | Almost | moderate | No | Mature |
| UT333 | J | 4 | F | 4 | 27 | Almost | 138 | Yes | 11.0 | Yes | moderate | No | Immature |
| UT275 | A | 3 | F | 1 | 44.4 | Yes | 144 | Yes | 18.3 | Yes | good | No | Mature |
| UT278 | J | 3 | F | 1 | 29.7 | Yes | 130 | Yes | 13.7 | Yes | moderate | No | Immature |
| UT332 | A | 8 | F | 2 | 54 | Yes | 156.5 | Yes | 23.0 | Yes | moderate | Yes | Mature |
| UT289 | A | 4 | F | 3 | 39 | No | 147 | Almost |  | No | good | Yes | Mature |
| UT288 | J |  | F | 2 |  | No | 122 | Almost |  | No | good | No | Immature |
| UT290 | A | 8 | F | 3 | 37 | Almost | 149 | Yes | 17.3 | Yes | moderate | Yes | Mature |
| UT291 | J | 2 | F | 2 | 28.5 | No | 125 | Yes | 24.7 | Yes | good | No | Immature |
| UT287 | J |  | F | 3 | 12.5 | No | 127.5 | Almost |  | No | good | No | Immature |
| UT282 | A | 9 | F | 1 | 60.5 | Yes | 162 | Yes | 24.3 | Yes | good | No | Mature |
| UT307 | A |  | F | 2 | 35 | Yes | 146 | Yes | 18.3 | Yes | moderate | No | Mature |
| UT391 | A | 7.5 | F | 2 | 24.5 | Yes | 136 | Yes | 15.3 | Yes | poor | No | Mature |
| UT381 | A |  | F | 3 | 36.5 | Yes | 150 | Yes | 15.7 | Yes | moderate | No | Mature |
| UT389 | A | 11 | F | 4 | 40 | No | 145 | Yes | 17.7 | Yes | moderate | Yes | Mature |
| UT346 | A | 7.5 | F | 2 | 50 | Yes | 148 | Yes | 16.7 | Yes | good | Yes | Mature |
| UT385 | A | 8 | F | 4 | 46 | No | 160 | Almost | 24.0 | Yes | good | Yes | Mature |
| UT401 | A |  | F | 2 | 31.5 | Yes | 149 | Yes | 8.0 | Yes | poor | No | Mature |
| UT357 | A | 11 | F | 2 | 50.8 | Yes | 161 | Yes | 19.3 | Yes | moderate | No | Mature |
| UT352 | A | 6 | F | 4 | 38.5 | Almost | 164 | Almost | 11.3 | Almost | moderate | No | Mature |
| UT340 | A | 11 | F | 1 | 41.6 | Yes | 151.5 | Yes | 14.3 | Yes | moderate | No | Mature |
| UT348 | A |  | F | 4 | 38 | Almost | 153 | Yes | 8.0 | Yes | moderate | No | Mature |
| UT347 | J | 2 | F | 2 | 24.5 | Almost | 131 | Yes | 13.0 | Yes | poor | No | Immature |
| UT666 | A |  | F | 4 | 27 | Almost | 150 | Almost | 6.0 | Almost | Unknown | No | Mature |
| UT363 | A | 9 | F | 4 | 41.5 | No | 160 | No |  | No | moderate | No | Mature |
| UT356 | J |  | F | 3 | 19.8 | No | 127 | Yes | 2.0 | Yes | moderate | No | Immature |
| UT667 | J |  | F | 3 | 28 | Yes | 125.5 | Yes | 15.0 | Almost | good | No | Immature |
| UT672 | J |  | F | 4 | 21 | Almost | 123 | Yes | 12.7 | Almost | moderate | No | Immature |
| UT372 | A | 9 | F | 1 | 53 | Yes | 150.5 | Yes | 21.0 | Yes | good | No | Mature |
| UT373 | A | 4 | F | 1 | 36.5 | Almost | 140 | Yes | 20.3 | Yes | moderate | No | Mature |
| UT379 | A | 6 | F | 2 | 43 | Yes | 160.5 | Yes | 12.3 | Yes | moderate | No | Mature |
| UT420 | A |  | F | 3 | 51.5 | Almost | 162.5 | Yes |  | No | Unknown | No | Mature |
| UT418 | A |  | F | 2 | 55.5 | Yes | 158 | Yes | 20.7 | Yes | moderate | No | Mature |
| UT673 | A |  | F | 4 | 36 | Yes | 152 | Almost | 5.0 | Almost | good | Yes | Mature |
| UT408 | J |  | F | 4 | 19 | No | 122 | Almost |  | No | moderate | No | Immature |
| UT410 | A | 18.5 | F | 2 | 48 | Yes | 166.5 | Yes | 15.0 | Yes | poor | No | Mature |
| UT417 | A | 9 | F | 4 | 38 | Almost | 149 | Almost | 20.0 | Almost | good | Yes | Mature |
| UT732 | A |  | F | 3 | 19.5 | No | 158 | Yes | 17.0 | Almost | good | Unknown | Mature |
| UT407 | A | 7 | F | 1 | 49 | Yes | 159.5 | Yes | 11.0 | Yes | poor | Yes | Mature |
| UT449 | J |  | F | 3 | 15.5 | No | 121.8 | Yes | 12.0 | Yes | poor | No | Immature |
| UT453 | J | 1.5 | F | 2 | 32.9 | Yes | 125.5 | Yes | 24.3 | Yes | good | No | Immature |
| UT447 | A |  | F | 2 | 39 | Yes | 149.5 | Yes | 13.0 | Yes | moderate | No | Mature |
| UT602 | A | 4 | F | 2 | 39.2 | Yes | 144 | Yes | 22.7 | Yes | good | No | Mature |
| UT678 | A | 9 | F | 4 | 49.5 | Yes | 151 | Yes | 23.7 | Almost | good | Yes | Mature |
| UT433 | A | 4 | F | 1 | 35 | Yes | 139 | Yes | 22.7 | Yes | good | No | Mature |
| UT439 | J | 5 | F | 4 | 20.5 | Yes | 130 | Yes | 15.7 | Yes | moderate | No | Immature |
| UT537 | A |  | F | 2 | 42.1 | Yes | 158 | Yes | 18.3 | Yes | moderate | No | Mature |
| UT555 | J |  | F | 3 | 16.3 | Yes | 121 | Yes | 6.0 | Almost | poor | No | Immature |
| UT481 | A |  | F | 2 | 37 | Yes | 152 | Yes | 6.7 | Yes | poor | No | Mature |
| UT480 | J |  | F | 3 | 19.5 | Almost | 122 | Yes | 6.0 | Almost | poor | No | Immature |
| UT479 | A | 8 | F | 4 | 27 | Almost | 143 | Yes | 8.3 | Almost | moderate | No | Mature |
| UT468 | A |  | F | 1 | 47.5 | Yes | 156 | Yes | 9.7 | Yes | poor | No | Mature |
| UT471 | A |  | F | 4 | 36.5 | Almost | 157.5 | Almost | 13.0 | Almost | moderate | No | Mature |
| UT571 | J |  | F | 4 | 19.8 | Almost | 124 | Yes | 6.0 | Almost | moderate | No | Immature |
| UT580 | A |  | F | 3 | 42 | Almost | 149 | Yes | 22.7 | Almost | good | No | Mature |
| UT904 | J |  | F | 4 | 18 | Almost | 127 | Almost |  | No | Unknown | No | Immature |
| UT594 | A | 5 | F | 4 | 32.2 | Almost | 162.5 | Yes |  | No | poor | No | Mature |
| UT591 | A | 8 | F | 4 | 33.6 | Almost | 142.5 | Yes | 18.0 | Almost | good | No | Mature |
| UT627 | J |  | F | 4 | 20.5 | Almost | 126.5 | Almost | 7.7 | Almost | poor | No | Immature |
| UT619 | J |  | F | 4 | 15.4 | Almost | 122 | Yes | 2.7 | Almost | moderate | No | Immature |
| UT562 | J |  | F | 4 | 18 | Almost | 124 | Yes | 2.3 | Almost | poor | No | Immature |
| UT516 | A | 7 | F | 4 | 54 | Yes | 164 | Yes | 11.7 | Almost | poor | No | Mature |
| UT620 | J |  | F | 4 | 16.3 | Yes | 123 | Yes | 5.0 | Almost | poor | No | Immature |
| UT760 | J | 1 | F | 2 | 20.1 | Yes | 123 | Yes | 5.3 | Yes | poor | No | Immature |
| UT798 | A |  | F | 4 | 42 | Almost | 147.5 | Yes |  | No | Unknown | No | Mature |
| UT800 | J |  | F | 4 | 20 | Yes | 122 | Yes |  | No | Unknown | No | Immature |
| UT596 | J |  | F | 4 | 24.2 | No | 128 | Yes | 14.3 | Almost | good | No | Immature |
| UT575 | A |  | F | 4 | 32.5 | Almost | 157 | Almost | 4.7 | Almost | good | No | Mature |
| UT565 | J |  | F | 4 | 20.9 | Yes | 124.5 | Yes | 17.0 | Almost | good | No | Immature |
| UT566 | A |  | F | 4 | 36.5 | Almost | 149 | Yes | 11.3 | Almost | moderate | No | Mature |
| UT529 | A | 6 | F | 2 | 27 | Yes | 135.5 | Yes | 6.0 | Yes | poor | No | Mature |
| UT522 | A | 4.5 | F | 1 | 36.8 | Yes | 153.5 | Yes | 12.7 | Yes | moderate | No | Mature |
| UT780 | A |  | F | 4 | 28 | No | 150 | No | 2.0 | Yes | moderate | No | Mature |
| UT583 | A |  | F | 4 | 39.5 | Almost | 158.5 | Yes | 5.0 | Almost | moderate | No | Mature |
| UT805 | A |  | F | 4 | 24.5 | Almost | 148 | Yes |  | No | Unknown | No | Mature |
| UT526 | A |  | F | 1 | 41 | Yes | 155 | Yes | 10.0 | Yes | poor | No | Mature |
| UT611 | A |  | F | 4 | 25.5 | Almost | 147 | Almost | 14.0 | Almost | good | No | Mature |
| UT622 | A |  | F | 3 | 34 | Yes | 158 | Yes | 11.0 | Yes | moderate | No | Mature |
| UT623 | A | 6 | F | 2 | 40 | Yes | 168.5 | Yes | 9.7 | Yes | poor | Yes | Mature |
| UT528 | A | 24 | F | 2 | 56 | Yes | 166 | Yes | 16.7 | Yes | moderate | No | Mature |
| UT613 | J |  | F | 4 | 16.5 | Almost | 128 | Yes | 8.0 | Almost | poor | No | Immature |
| UT1332 | A | 7 | F | 2 | 62 | Yes | 154 | Yes | 15.0 | Yes | good | Yes | Mature |
| UT1328 | A |  | F | 4 | 45.5 | Yes | 164 | Yes | 12.3 | Yes | moderate | No | Mature |
| UT1337 | A | 8 | F | 3 | 43 | Yes | 155 | Yes | 9.3 | Yes | poor | Yes | Mature |
| UT660 | J | 2 | F | 3 | 17 | No | 121 | Yes | 27.3 | Yes | good | No | Immature |
| UT1338 | A |  | F | 3 | 42 | Yes | 156 | Yes | 17.3 | Yes | good | Unknown | Mature |
| UT837 | A |  | F | 4 | 44.5 | Yes | 154.5 | Yes | 20.0 | Yes | moderate | No | Mature |
| UT1336 | J |  | F | 3 | 28 | Yes | 126 | Yes | 15.3 | Yes | moderate | No | Immature |
| UT838 | J |  | F | 4 | 22.3 | No | 125.3 | Yes | 21.7 | Yes | moderate | No | Immature |
| UT1333 | A | 8 | F | 2 | 61 | Yes | 155 | Yes | 18.3 | Yes | good | Yes | Mature |
| UT1326 | A | 6 | F | 4 | 38 | Almost | 159 | Yes | 9.3 | Yes | poor | Yes | Mature |
| UT829 | J |  | F | 4 | 25.8 | No | 129 | No | 17.3 | Almost | Unknown | No | Immature |
| UT1310 | A |  | F | 3 | 41.3 | Almost | 148.5 | Yes | 18.7 | Yes | poor | No | Mature |
| UT544 | A |  | F | 1 | 45 | Yes | 157.5 | Yes | 19.0 | Yes | moderate | No | Mature |
| UT841 | A |  | F | 2 | 39.8 | Yes | 151 | Yes | 13.3 | Yes | poor | No | Mature |
| UT564 | A | 10 | F | 2 | 43 | Yes | 153 | Yes | 17.7 | Yes | moderate | No | Mature |
| UT857 | J |  | F | 3 | 22.2 | Almost | 125 | Yes | 21.3 | Yes | moderate | No | Immature |
| UT1268 | A | 15 | F | 2 | 42 | Yes | 138 | Yes | 9.3 | Yes | moderate | Yes | Mature |
| UT1269 | J |  | F | 2 | 30.5 | Yes | 132.5 | Yes | 18.7 | Yes | moderate | No | Immature |
| UT1267 | A | 5 | F | 2 | 37 | No | 149 | Yes | 20.0 | No | good | No | Mature |
| UT694 | A |  | F | 3 | 35 | No | 155.5 | Almost |  | No | good | No | Mature |
| UT703 | J | 1 | F | 3 | 12.5 | No | 132 | Yes |  | No | moderate | No | Immature |
| UT716 | J |  | F | 2 | 18.5 | No | 123 | Yes | 16.0 | Almost | moderate | No | Immature |
| UT717 | J |  | F | 3 | 5 | No | 121 | Almost |  | No | Unknown | No | Immature |
| UT718 | J |  | F | 4 | 7.5 | No | 126 | Almost |  | No | Unknown | No | Unknown |
| UT860 | A | 15 | F | 4 | 33.5 | Almost | 142 | Almost | 19.7 | Almost | moderate | No | Mature |
| UT833 | A |  | F | 4 | 30.6 | Almost | 154 | Almost |  | No | Unknown | NE | Mature |
| UT1319 | A | 5 | F | 4 | 37.5 | Yes | 147 | Yes | 16.3 | Yes | moderate | No | Mature |
| UT810 | A |  | F | 4 | 24.5 | No | 150 | No | 21.3 | Almost | good | No | Mature |
| UT873 | A |  | F | 4 | 25 | Almost | 146.5 | Almost |  | No | Unknown | NE | Mature |
| UT899 | A |  | F | 4 | 40.5 | Yes | 146 | Yes | 10.0 | Almost | poor | No | Mature |
| UT900 | A |  | F | 4 | 36 | Almost | 143 | Almost | 14.3 | Almost | moderate | NE | Mature |
| UT852 | J |  | F | 4 | 18.4 | Yes | 124.5 | Yes | 9.0 | Almost | moderate | No | Immature |
| UT1323 | A | 9 | F | 4 | 43 | Almost | 133 | No | 12.0 | No | moderate | No | Mature |
| UT842 | J |  | F | 4 | 17.2 | Almost | 123 | Yes | 5.0 | Almost | Unknown | No | Immature |
| UT881 | J |  | F | 4 | 17.5 | Almost | 123 | Almost |  | No | Unknown | No | Immature |
| UT898 | J |  | F | 4 | 15.7 | Almost | 121 | Almost |  | No | Unknown | No | Immature |
| UT775 | A | 7 | F | 1 | 31.5 | Yes | 137 | Yes | 12.7 | Yes | poor | No | Mature |
| UT781 | A |  | F | 4 | 43 | Yes | 159 | Yes | 18.0 | Yes | moderate | NE | Mature |
| UT1271 | A | 6 | F | 3 | 42.5 | Almost | 132 | No | 13.3 | Yes | good | Yes | Mature |
| UT895 | A |  | F | 4 | 33 | Almost | 144 | Almost | 12.7 | Almost | Unknown | NE | Mature |
| UT896 | J |  | F | 4 | 16 | Almost | 124 | Almost | 8.0 | Almost | Unknown | No | Immature |
| UT1022 | A |  | F | 2 | 43.5 | Yes | 151 | Yes | 10.0 | Yes | moderate | No | Mature |
| UT1019 | A | 5.5 | F | 2 | 46 | Yes | 149 | Yes | 15.0 | Yes | moderate | Yes | Mature |
| UT1325 | A | 6 | F | 3 | 53.5 | Yes | 161.5 | Yes | 19.0 | Yes | good | Yes | Mature |
| UT1279 | A | 8 | F | 2 | 57.5 | Yes | 160 | Yes | 19.3 | Yes | moderate | No | Mature |
| UT1284 | A | 8 | F | 3 | 44 | Yes | 152 | Yes | 15.7 | Yes | good | No | Mature |
| UT1320 | A | 6 | F | 4 | 33 | No | 145 | Yes | 10.0 | No | Unknown | Yes | Mature |
| UT917 | J | 1 | F | 2 | 22 | Yes | 122.6 | Yes | 13.3 | Yes | poor | No | Immature |
| UT1278 | A | 13 | F | 3 | 51 | Yes | 162 | Yes | 9.7 | Yes | poor | No | Mature |
| UT937 | A | 6.5 | F | 3 | 35 | No | 142 | Yes |  | No | good | Yes | Mature |
| UT952 | A |  | F | 3 | 44 | Yes | 142 | Yes | 11.0 | Yes | poor | NE | Mature |
| UT955 | A | 5 | F | 2 | 50 | Almost | 148 | Yes | 14.3 | Yes | moderate | Yes | Mature |
| UT956 | J | 1 | F | 2 | 26 | Yes | 126 | Yes | 14.0 | Yes | poor | No | Immature |
| UT1281 | A | 11 | F | 3 | 39 | Yes | 145.5 | Yes | 10.0 | Yes | poor | No | Mature |
| UT1266 | A | 4.5 | F | 2 | 47 | Yes | 148 | Yes | 15.3 | Yes | good | Yes | Mature |
| UT975 | A |  | F | 2 | 49 | Yes | 159.5 | Yes | 11.7 | Yes | poor | No | Mature |
| UT988 | A |  | F | 3 | 42 | Almost | 161 | Yes | 11.0 | Yes | moderate | No | Mature |
| UT981 | A |  | F | 4 | 29.2 | No | 150 | Almost | 10.0 | Almost | moderate | Yes | Mature |
| UT995 | A | 11 | F | 4 | 32.5 | Yes | 165 | Yes | 22.7 | Yes | good | Unknown | Mature |
| UT990 | A | 5 | F | 4 | 30.3 | Yes | 135.5 | Yes | 12.7 | Yes | moderate | No | Mature |
| UT992 | A |  | F | 4 | 32.7 | Yes | 145 | Almost | 14.0 | Almost | Unknown | No | Mature |
| UT1001 | A |  | F | 4 | 48 | Yes | 159 | Yes | 9.0 | Almost | good | No | Mature |
| UT1285 | A | 5 | F | 4 | 42.5 | Yes | 148 | Yes | 15.7 | Yes | good | No | Mature |
| UT1282 | A |  | F | 4 | 26.5 | No | 146 | Yes | 2.0 | Almost | Unknown | No | Mature |
| UT1287 | A |  | F | 4 | 37.5 | Almost | 151.5 | Yes | 9.3 | Yes | moderate | No | Mature |
| UT1288 | A | 8 | F | 4 | 40 | Almost | 154.5 | Yes | 8.7 | Yes | Unknown | No | Mature |
| UT1006 | A | 10 | F | 1 | 38.5 | Yes | 141 | Yes | 16.7 | Yes | good | Yes | Mature |
| UT1322 | J | 4 | F | 4 | 33.5 | Yes | 142 | No | 10.0 | No | moderate | No | Immature |
| UT1286 | A | 7 | F | 4 | 34 | Almost | 158 | Yes | 8.7 | No | Unknown | No | Mature |
| UT1005 | A | 9.5 | F | 1 | 41.7 | Yes | 147 | Yes | 10.3 | Yes | poor | No | Mature |
| UT1007 | A | 5 | F | 1 | 42 | Almost | 144 | Yes | 16.3 | Yes | moderate | No | Mature |
| UT1304 | A | 6 | F | 4 | 55 | Yes | 155 | Yes | 14.0 | Yes | moderate | Yes | Mature |
| UT1020 | J | 3 | F | 1 | 27.5 | Yes | 130 | Yes | 21.3 | Yes | moderate | No | Immature |
| UT1302 | J | 2 | F | 2 | 39.8 | Almost | 133.5 | Yes | 29.0 | Yes | good | No | Immature |
| UT1311 | A | 5 | F | 2 | 53.5 | No | 158 | Yes | 20.0 | No | good | Yes | Mature |
| UT1312 | J | 2 | F | 1 | 33 | No | 131.5 | No | 24.0 | Yes | good | No | Immature |
| UT1460 | A |  | F | 3 | 42.5 | Yes | 146 | Yes | 13.3 | Yes | moderate | No | Mature |
| UT1316 | A | 6.5 | F | 1 | 47.5 | Yes | 153 | Yes | 11.0 | Yes | poor | No | Mature |
| UT1419 | A | 8.5 | F | 1 | 48.5 | Yes | 159 | Yes | 8.3 | Yes | poor | No | Mature |
| UT1500 | A | 6.5 | F | 4 | 29 | Yes | 152 | Yes |  | No | Unknown | No | Mature |
| UT1418 | A | 8 | F | 2 | 53.5 | Yes | 151 | Yes | 14.0 | Yes | moderate | Yes | Mature |
| UT1448 | A | 4 | F | 2 | 47.5 | Yes | 153 | Yes | 24.7 | Yes | good | No | Mature |
| UT1473 | A | 8 | F | 4 | 48 | Yes | 171 | Yes | 6.0 | Yes | poor | No | Mature |
| UT1470 | A | 8.5 | F | 2 | 38 | Yes | 152.5 | Yes | 12.3 | Yes | poor | No | Mature |
| UT1480 | J | 5 | F | 2 | 23.2 | Yes | 131.5 | Yes | 8.0 | Yes | poor | No | Immature |
| UT1482 | A | 7 | F | 2 | 33.5 | Yes | 155 | Yes | 9.3 | Yes | poor | No | Mature |
| UT1484 | A | 8 | F | 1 | 40.4 | Yes | 164 | Yes | 9.0 | Yes | poor | Yes | Mature |
| UT1496 | A | 4 | F | 3 | 45 | No | 150 | No |  | No | good | Yes | Mature |
| UT1503 | J | 3 | F | 3 | 28 | No | 122 | Yes | 20.0 | Yes | good | No | Immature |
| UT1508 | A | 11 | F | 2 | 58 | Yes | 158 | Yes | 24.0 | Yes | good | No | Mature |
| UT1543 | A | 7 | F | 4 | 43 | Almost | 152 | No | 10.0 | Yes | poor | No | Mature |
| UT1530 | A | 8 | F | 3 | 31.5 | Almost | 149 | Yes |  | No | poor | No | Mature |
| UT1527 | A | 8 | F | 1 | 39 | Yes | 157 | Yes | 7.7 | Yes | poor | No | Mature |
| UT1535 | A | 6 | F | 1 | 46 | Yes | 146 | Yes | 7.0 | Yes | poor | No | Mature |
| UT1537 | J | 3.5 | F | 2 | 21.5 | Yes | 129 | Yes | 8.7 | Yes | poor | No | Immature |
| UT1551 | A | 10 | F | 3 | 47 | Yes | 151 | Yes | 10.3 | Yes | moderate | Yes | Mature |
| UT1565 | J |  | F | 4 | 21 | Almost | 122 | Yes | 13.0 | Yes | moderate | No | Immature |
| UT1555 | A | 8 | F | 2 | 50 | Almost | 150 | Yes | 15.0 | Yes | good | Yes | Mature |
| UT1572 | A | 6 | F | 1 | 63.5 | Yes | 152.5 | Yes | 23.3 | Yes | good | Yes | Mature |
| UT1576 | J |  | F | 2 | 27.5 | Yes | 134 | Yes | 15.3 | Yes | poor | No | Immature |
| UT1577 | A | 12 | F | 1 | 43 | Yes | 149 | Yes | 16.3 | Yes | moderate | No | Mature |
| UT1579 | A | 8 | F | 1 | 53 | Almost | 133 | Yes | 25.7 | Yes | good | Yes | Mature |
| UT1581 | A | 6 | F | 2 | 60.5 | Yes | 139 | Yes | 18.3 | Yes | good | Yes | Mature |
| UT1582 | A | 9.5 | F | 1 | 44 | Yes | 149 | Yes | 10.0 | Yes | moderate | No | Mature |
| UT1603 | A |  | F | 4 | 30 | No | 153 | Almost | 8.0 | No | poor | Unknown | Mature |
| UT1613 | J | 3 | F | 3 | 28 | almost | 128 | Yes | 7.0 | Yes | poor | No | Immature |
| UT1604 | A | 8 | F | 3 | 32 | Yes | 148 | Yes | 9.3 | Yes | poor | No | Mature |
| UT1629 | J |  | F | 4 | 20 | No | 125 | Yes |  | No | Unknown | No | Immature |
| UT1611 | A | 12 | F | 1 | 44.5 | Yes | 167 | yes | 10.7 | Yes | poor | No | Mature |
| UT1642 | A | 7 | F | 4 | 35 | Almost | 156 | Yes |  | No | Unknown | Yes | Mature |
| UT1614 | A | 6 | F | 3 | 37.5 | Almost | 155 | Yes | 5.7 | Yes | poor | No | Mature |
| UT1636 | A | 11 | F | 2 | 38 | Yes | 156 | Yes | 5.0 | Yes | poor | Yes | Mature |
| UT1638 | A | 6 | F | 2 | 35.5 | Yes | 153 | Yes | 5.0 | Yes | poor | No | Mature |
| UT1631 | A | 7.5 | F | 3 | 43 | No | 159 | Yes | 10.7 | No | moderate | Yes | Mature |
| UT1624 | A | 8 | F | 3 | 43 | Almost | 161 | Yes | 8.7 | Yes | poor | No | Mature |
| UT1632 | A |  | F | 3 | 38 | No | 147 | Yes |  | No | Unknown | No | Mature |
| UT1644 | A | 8 | F | 1 | 46 | Yes | 147 | Yes | 13.7 | Yes | good | No | Mature |
| UT1645 | A | 6 | F | 2 | 42.5 | Almost | 157 | Yes | 11.7 | Yes | moderate | No | Mature |
| UT1668 | A | 10 | F | 4 | 28.5 | No | 143 | Approx. | 26.0 | Approx. | good | No | Mature |
| UT1663 | A | 7.5 | F | 4 | 52 | Almost | 151 | Yes | 12.0 | Almost | moderate | Yes | Mature |
| UT1683 | A |  | F | 4 | 31 | No | 137.5 | Yes |  | No | Unknown | No | Mature |
| UT1687 | A | 8 | F | 3 | 40.5 | Almost | 149 | Yes | 13.3 | Yes | moderate | No | Mature |
| UT1710 | J | 3 | F | 3 | 27 | Real | 135 | Yes | 3.7 | Yes | poor | No | Immature |
| UT1720 | A | 9 | F | 3 | 42 | No | 165 | Approx | 22.0 | No | good | Yes | Mature |
| UT1717 | A | 8.5 | F | 2 | 37 | Almost | 149 | Yes | 12.7 | Yes | poor | No | Mature |
| UT1724 | J | 3.5 | F | 3 | 37.4 | Almost | 132 | Yes | 11 | Yes | poor | No | Immature |
| UT1727 | J | 5 | F | 2 | 37 | Almost | 141 | Yes | 24.7 | Yes | good | No | Immature |
| UT1729 | A | 6.5 | F | 2 | 50.5 | Yes | 145 | Yes | 19.7 | Yes | good | Yes | Mature |
| UT1728 | A | 8.5 | F | 1 | 64.5 | Yes | 155 | Yes | 22.7 | Yes | good | Yes | Mature |
| UT1747 | A | 5 | F | 2 | 60 | Almost | 161.5 | Yes | 20.3 | Yes | moderate | Yes | Mature |
| UT1750 | J | 3 | F | 2 | 29.7 | Yes | 131.5 | Yes | 14 | Yes | moderate | No | Immature |
| UT1752 | A | 11 | F | 2 | 42.5 | Yes | 151.5 | Yes | 21.3 | Yes | moderate | No | Mature |
| UT1753 | A | 7 | F | 2 | 55.5 | Yes | 147.5 | Yes | 14.3 | Yes | poor | Yes | Mature |
| UT1756 | A | 8 | F | 2 | 59.5 | Yes | 151 | Yes | 16.7 | Yes | moderate | Yes | Mature |
| UT1760 | A | 5 | F | 4 | 40 | Yes | 148.5 | Yes | 13.3 | Yes | poor | No | Mature |
| UT1761 | A | 8 | F | 1 | 59 | Yes | 175 | Yes | 18.3 | Yes | good | No | Mature |
| UT1767 | A | 17 | F | 3 | 34 | Real | 150 | Yes | 9.3 | Yes | poor | No | Mature |
| UT1771 | A |  | F | 4 | 42 | Almost | 155 | Yes | 8 | Approx. | poor | Possible | Mature |
| UT1776 | A |  | F | 3 | 34.5 | Almost | 145.5 | Yes | 9 | Yes | poor | Yes | Mature |
| UT1777 | A |  | F | 3 | 30.5 | Almost | 157 | Yes | 4.3 | Approx. | poor | No | Mature |
| UT1778 | A |  | F | 4 | 40 | No | 168 | Almost | 7.5 | Approx. | poor | No | Mature |
| UT1782 | A | 7.5 | F | 2 | 48.5 | Almost | 146 | Yes | 16.3 | Yes | good | Yes | Mature |
| TX044 | J | 2 | F | 1 | 25 | Yes | 119 | Yes | 17.7 | Yes | good | No | Immature |
| UT034 | J |  | F | 4 | 29 | Almost | 120 | Almost | 37.7 | Yes | good | No | Immature |
| UT055 | J |  | F | 2 | 19 | Yes | 116 | Yes | 9.7 | Yes | moderate | No | Immature |
| UT113 | J |  | F | 1 | 24.3 | Yes | 117 | Yes | 11.7 | Yes | moderate | No | Immature |
| UT165 | J |  | F | 4 | 18.3 | No | 117 | Yes | 8.0 | No | poor | No | Immature |
| UT196 | J |  | F | 2 | 26 | Yes | 119 | Yes | 21.0 | Yes | good | No | Immature |
| UT211 | J |  | F | 2 | 32 | Almost | 119 | Yes | 25.7 | Yes | good | No | Immature |
| UT247 | J |  | F | 4 | 15 | No | 120 | No |  | No | Unknown | No | Immature |
| UT268 | J |  | F | 2 | 23 | Yes | 119 | Yes | 15.0 | Yes | moderate | No | Immature |
| UT274 | J |  | F | 2 | 26 | Almost | 117.5 | Yes | 14.0 | Yes | moderate | No | Immature |
| UT314 | J |  | F | 4 | 26.5 | No | 119 | Yes | 17.0 | Yes | moderate | No | Immature |
| UT294 | J |  | F | 2 | 38 | Yes | 120 | Yes | 25.3 | Yes | good | No | Immature |
| UT384 | J |  | F | 3 | 22.5 | Yes | 116 | Yes | 12.3 | Yes | moderate | No | Immature |
| UT397 | J |  | F | 4 | 16 | No | 120 | No | 15.7 | Yes | moderate | No | Immature |
| UT421 | J | 1 | F | 1 | 24.5 | Yes | 117 | Yes | 20.3 | Yes | good | No | Immature |
| UT429 | J |  | F | 3 | 21.5 | Yes | 118 | Yes | 7.3 | Yes | poor | No | Immature |
| UT442 | J |  | F | 2 | 18.2 | Yes | 119 | Yes | 7.3 | Yes | poor | No | Immature |
| UT474 | J |  | F | 4 | 18.5 | Almost | 119.7 | Yes | 2.0 | Almost | poor | No | Immature |
| UT510 | J |  | F | 4 | 18 | Almost | 117 | Yes |  | No | Unknown | No | Immature |
| UT511 | J |  | F | 4 | 16.5 | Almost | 117.5 | Yes | 3.0 | Almost | Unknown | No | Immature |
| UT547 | J |  | F | 4 | 12.8 | Almost | 119 | Yes | 3.0 | Almost | poor | No | Immature |
| UT554 | J |  | F | 4 | 13.6 | Almost | 118 | Almost | 2.0 | Almost | Unknown | No | Immature |
| UT570 | J |  | F | 4 | 15.5 | Yes | 117 | Yes | 2.7 | Almost | poor | No | Immature |
| UT574 | J |  | F | 3 | 17.5 | Yes | 117.5 | Yes |  | No | Unknown | No | Immature |
| UT608 | J |  | F | 4 | 21.6 | Almost | 120 | Yes | 11.7 | Almost | moderate | No | Immature |
| UT648 | J |  | F | 4 | 13.9 | Almost | 118.5 | Yes | 3.3 | Almost | moderate | No | Immature |
| UT650 | J |  | F | 4 | 24 | Yes | 117.5 | Yes | 26.3 | Almost | good | No | Immature |
| UT657 | J |  | F | 4 | 23.4 | Almost | 117.5 | Yes | 18.0 | Almost | good | No | Immature |
| UT665 | J |  | F | 3 | 22.9 | Yes | 120 | Yes | 21.7 | Almost | moderate | No | Immature |
| UT754 | J |  | F | 4 | 13.75 | No | 120 | Almost |  | No | Unknown | No | Immature |
| UT761 | J |  | F | 4 | 15.5 | Almost | 119 | Yes | 3.0 | Almost | poor | No | Immature |
| UT803 | J |  | F | 4 | 19.5 | Yes | 118.3 | Yes |  | No | Unknown | No | Immature |
| UT927 | J |  | F | 4 | 13.5 | Almost | 118 | No | 3.0 | Almost | Unknown | No | Immature |
| UT1029 | J |  | F | 3 | 25.5 | Yes | 116.5 | Yes | 23.7 | Yes | good | No | Immature |
| UT737 | J |  | F | 3 | 16.5 | Almost | 117 | Yes | 6.7 | Almost | poor | No | Immature |
| UT784 | J |  | F | 4 | 15.5 | Yes | 120 | Yes | 2.0 | Almost | Unknown | No | Immature |
| UT821 | J |  | F | 4 | 15 | Almost | 116.5 | Almost | 12.7 | Almost | Unknown | No | Immature |
| UT831 | J |  | F | 4 | 16 | Almost | 118.5 | Almost | 5.0 | Almost | Unknown | No | Immature |
| UT888 | J |  | F | 4 | 13 | Almost | 117 | Almost | 3.7 | Almost | Unknown | No | Immature |
| UT1008 | J | 2 | F | 3 | 19.8 | Yes | 120 | Yes | 7.7 | Yes | poor | No | Immature |
| UT1013 | J |  | F | 4 | 15.6 | Yes | 120 | Yes | 3.3 | Yes | poor | No | Immature |
| UT855 | J | 1 | F | 1 | 25.5 | Yes | 116 | Yes | 30.0 | Yes | good | No | Immature |
| UT1444 | J |  | F | 2 | 15.4 | No | 117 | Yes |  | No | good | NE | Unknown |
| UT1478 | J |  | F | 3 | 17.4 | Yes | 118.4 | Yes | 3.7 | Yes | poor | No | Immature |
| UT1505 | J |  | F | 2 | 25 | No | 116 | Yes | 20.3 | Yes | good | No | Immature |
| UT1521 | J |  | F | 2 | 23 | Yes | 119 | Yes | 17.3 | Yes | good | No | Immature |
| UT1538 | J |  | F | 2 | 12 | No | 116 | No |  | No | Unknown | No | Immature |
| UT1567 | J |  | F | 3 | 21 | Almost | 119 | Yes | 8.7 | Yes | poor | No | Immature |
| UT1587 | J |  | F | 3 | 28 | Almost | 116 | Yes | 24.7 | Yes | good | NE | Unknown |
| UT1588 | J |  | F | 3 | 28 | Almost | 118 | Yes | 24.3 | Yes | good | No | Immature |
| UT1618 | J |  | F | 4 | 13.5 | no | 116 | No |  | No | Unknown | No | Immature |
| UT1655 | J |  | F | 2 | 25 | No | 116 | Yes | 21.7 | Yes | good | No | Immature |
| UT1665 | J |  | F | 2 | 25.3 | Almost | 119 | Yes | 14.0 | Yes | moderate | No | Immature |
| UT1670 | J |  | F | 2 | 25 | Unknown | 115.5 | Yes | 17.0 | Yes | good | No | Immature |
| UT1678 | J |  | F | 4 | 14.5 | No | 119 | Approx. |  | No | Unknown | No | Immature |
| UT1700 | J |  | F | 2 | 17.5 | Almost | 116 | Yes | 9.0 | Yes | poor | No | Immature |
| UT1439 | A |  | F | 4 | 31.5 | Almost | 151 | Yes |  | No | Unknown | NE | Mature |
| UT1440 | A |  | F | 4 | 36.5 | Almost | 154 | Yes |  | No | Unknown | NE | Mature |
| UT1441 | A |  | F | 3 | 41 | Almost | 161 | Yes |  | No | moderate | NE | Mature |
| UT1445 | A |  | F | 3 | 38.3 | Yes | 154 | Yes |  | No | poor | NE | Mature |
| UT1446 | A |  | F | 3 | 40.5 | Yes | 157 | Yes |  | No | moderate | NE | Mature |
| UT1459 | A |  | F | 4 |  | No | 148 | Almost |  | No | Unknown | NE | Mature |

***Supplementary Table 14.*** *Case information of harbour porpoise foetuses.*

| **ID** | **Sex** | **TL** | **TL_real** | **Single** |
| --- | --- | --- | --- | --- |
| TX037.1 | M | 28 | Yes | Yes |
| UT052.1 | M | 22 | Approx. | Yes |
| UT126.1 | M | 75 | Approx. | Yes |
| UT179.1 | U | 2.5 | Yes | Yes |
| UT332.1 | M | 28 | Yes | Yes |
| UT289.1 | M | 32 | Approx. | Yes |
| UT290.1 | F | 34 | Approx. | Yes |
| UT389.1 | F | 65 | Yes | Yes |
| UT346.1 | M | 68 | Yes | Yes |
| UT385.1 | F | 75 | Yes | Yes |
| UT673.1 | M | 23 | Yes | Yes |
| UT417.1 | M | 23 | Yes | Yes |
| UT407.1 | F | 41 | Yes | Yes |
| UT678.1 | F | 47 | Yes | No |
| UT678.2 | F | 49 | Yes | No |
| UT623.1 | M | 16 | Yes | Yes |
| UT1374.1 | F | 13 | Yes | Yes |
| UT1332.1 | M | 19 | Yes | Yes |
| UT1337.1 | F | 23 | Yes | Yes |
| UT1373.1 | F | 28 | Yes | Yes |
| UT1333.1 | M | 34 | Yes | Yes |
| UT1326.1 | M | 28 | Yes | Yes |
| UT1360.1 | M | 49 | Yes | Yes |
| UT1268.1 | F | 43 | Yes | Yes |
| UT1271.1 | F | 7 | Yes | Yes |
| UT1388.1 | F | 23.5 | Yes | Yes |
| UT1019.1 | M | 28 | Yes | Yes |
| UT1325.1 | M | 36 | Yes | Yes |
| UT1320.1 | F | 39.5 | Yes | Yes |
| UT938 | M | 62.3 | Yes | Yes |
| UT955.1 | F | 71.2 | Yes | Yes |
| UT1266.1 | M | 68 | Yes | Yes |
| UT964 | M | 62 | Yes | Yes |
| UT1006.1 | U | 1.5 | Yes | Yes |
| UT1304.1 | F | 11 | Approx. | Yes |
| UT1418.1 | F | 32 | Yes | Yes |
| UT1484.1 | U | 1 | Approx. | Yes |
| UT1496.1 | M | 50 | Yes | Yes |
| UT1551.1 | M | 11.3 | Yes | Yes |
| UT1555.1 | F | 32 | Yes | Yes |
| UT1572.1 | M | 64 | Yes | Yes |
| UT1579.1 | M | 67 | Yes | Yes |
| UT1581.1 | M | 73 | Yes | Yes |
| UT1642.1 | U | 2 | Approx. | Yes |
| UT1636.1 | F | 12 | Approx. | Yes |
| UT1631.1 | M | 18 | Yes | Yes |
| UT1663.1 | F | 73 | Yes | Yes |
| UT1720.1 | M | 31 | Yes | Yes |
| UT1729.1 | M | 43 | Yes | Yes |
| UT1747.1 | M | 56.5 | Yes | Yes |
| UT1753.1 | F | 72 | Yes | Yes |
| UT1756.1 | F | 76.5 | Yes | Yes |
| UT1776.1 | M | 8.7 | Yes | Yes |
| UT1782.1 | F | 31 | Yes | Yes |

***Supplementary Table 15.*** *Borders of study areas for the cumulative human impact assessment, based on the standard georeferenced marine names as published under the Flanders Marine Institute.*

| **ID** | **Study area** | **Layer** | **Name_first** | **Name_second** | **id** | **name** | **X** | **mrgid** |
| --- | --- | --- | --- | --- | --- | --- | --- | --- |
| 1 | Celtic & Irish waters | MarineRegions:iho | MarineRegions | iho | Iho.80 | Celtic Sea | include | 2351 |
| 1 | Celtic & Irish waters | MarineRegions:iho | MarineRegions | iho | iho.33 | Irish Sea and St. George's Channel | include | 2357 |
| 1 | Celtic & Irish waters | x_coords <- c(-13.0,-3.0,-3.0,-13.0, -13.0)  y_coords <- c(50.0,50.0,46.0,46.0, 50.0) | | | | | exclude |  |
| 2 | Kattegat Skagerrak Seas | MarineRegions:iho | MarineRegions | iho | Iho.96 | Kattegat | include | 2374 |
| 2 | Kattegat Skagerrak Seas | MarineRegions:iho | MarineRegions | iho | iho.98 | Skagerrak | include | 2379 |
| 3 | German North Sea | MarineRegions:eez | MarineRegions | eez | eez.218 | German Exclusive Economic Zone | include | 5669 |
| 3 | German North Sea | MarineRegions:iho | MarineRegions | iho | iho.57 | Baltic Sea | exclude | 2401 |
| 4 | Danish waters | MarineRegions:eez | MarineRegions | eez | eez.219 | Danish Exclusive Economic Zone | include | 5674 |
| 5 | Dutch waters | MarineRegions:eez | MarineRegions | eez | eez.217 | Dutch Exclusive Economic Zone | include | 5668 |
| 6 | Eastern Newfoundland | MarineRegions:lme | MarineRegions | lme | lme.5 | Labrador - Newfoundland | include | 8544 |
| 7 | English North Sea | MarineRegions:iho | MarineRegions | iho | iho.32 | Bristol Channel | include | 3141 |
| 7 | English North Sea | MarineRegions:iho | MarineRegions | iho | iho.85 | English Channel | include | 2389 |
| 7 | English North Sea | MarineRegions:iho | MarineRegions | iho | iho.88 | North Sea | include | 2350 |
| 7 | English North Sea | x_coords <- c(-8.034351,8.40,8.40,-8.034351, -8.034351)  y_coords <- c(55.811086,55.811086,63.0,63.0, 55.811086) | | | | | exclude |  |
| 7 | English North Sea | MarineRegions:eez | MarineRegions | eez | eez.217 | Dutch Exclusive Economic Zone | exclude | 5668 |
| 7 | English North Sea | MarineRegions:eez | MarineRegions | eez | eez.219 | Danish Exclusive Economic Zone | exclude | 5674 |
| 7 | English North Sea | MarineRegions:eez | MarineRegions | eez | eez.216 | Belgian Exclusive Economic Zone | exclude | 3293 |
| 7 | English North Sea | MarineRegions:eez_iho | MarineRegions | iho | iho.363 | Norwegian part of the North Sea | exclude | 5686 |
| 7 | English North Sea | MarineRegions:eez | MarineRegions | eez | eez.218 | German Exclusive Economic Zone | exclude | 5669 |
| 8 | Baltic Sea | MarineRegions:iho | MarineRegions | iho | iho.57 | Baltic Sea | include | 2401 |
| 9 | Gulf of Maine | World:gulf_of_maine | World | gulf_of_maine |  | Gulf of Maine | include | 8940 |
| 9 | Gulf of Maine | MarineRegions:iho | MarineRegions | iho | iho.19 | Bay of Fundy | exclude | 4289 |
| 10 | Bay of Fundy | MarineRegions:iho | MarineRegions | iho | iho.19 | Bay of Fundy | include | 4289 |
| 11 | Icelandic waters | MarineRegions:eez | MarineRegions | eez | eez.85 | Icelandic Exclusive Economic Zone | include | 5680 |
| 12 | Norwegian waters | MarineRegions:iho | MarineRegions | iho | iho.99 | Norwegian Sea | include | 2353 |
| 13 | Iberian Peninsula | MarineRegions:iho | MarineRegions | iho | iho.79 | Bay of Biscay | include | 2359 |
| 13 | Iberian Peninsula | MarineRegions:eez | MarineRegions | eez | eez.105 | Portuguese Exclusive Economic Zone | include | 5688 |
| 13 | Iberian Peninsula | MarineRegions:eez_iho | MarineRegions | eez_iho | iho.491 | Spanish part of the North Atlantic Ocean | include | 25366 |
| 13 | Iberian Peninsula | MarineRegions:eez_iho | MarineRegions | iho | iho.427 | Portuguese (Azores) part of the North Atlantic Ocean | exclude | 25318 |
| 13 | Iberian Peninsula | MarineRegions:eez_iho | MarineRegions | iho | iho.428 | Portuguese (Madeira) part of the North Atlantic Ocean | exclude | 25320 |
| 14 | Salish Sea | MarineRegions:iho | MarineRegions | iho | iho.74 | The Coastal Waters of Southeast Alaska and British Columbia | include | 4313 |
| 15 | Scottish waters | MarineRegions:iho | MarineRegions | iho | iho.34 | Inner Seas off the West Coast of Scotland | include | 4283 |
| 15 | Scottish waters | MarineRegions:iho | MarineRegions | iho | iho.88 | North Sea | include | 2350 |
| 15 | Scottish waters | x_coords <- c(-8.034351,-5.0,-3.0,8.40,8.40,-8.034351, -8.034351)  y_coords <- c(56.0,54.8,55.811086,55.811086,50.0,50.0, 56.0) | | | | | exclude |  |
| 15 | Scottish waters | MarineRegions:eez | MarineRegions | eez | eez.217 | Dutch Exclusive Economic Zone | exclude | 5668 |
| 15 | Scottish waters | MarineRegions:eez | MarineRegions | eez | eez.219 | Danish Exclusive Economic Zone | exclude | 5674 |
| 15 | Scottish waters | MarineRegions:eez | MarineRegions | eez | eez.216 | Belgian Exclusive Economic Zone | exclude | 3293 |
| 15 | Scottish waters | MarineRegions:eez_iho | MarineRegions | iho | iho.363 | Norwegian part of the North Sea | exclude | 5686 |
| 15 | Scottish waters | MarineRegions:eez | MarineRegions | eez | eez.218 | German Exclusive Economic Zone | exclude | 5669 |
| 15 | Scottish waters | MarineRegions:eez_iho | MarineRegions | iho | iho.537 | United Kingdom part of the North Atlantic Ocean | include | 25362 |
| 15 | Scottish waters | x_coords <- c(-2.0,-6.26,-7.18,-9.29, -10.63, -17.13, -3.42, -0.88, -2.0)  y_coords <- c(61.0,58.52,57.52,47.04, 47.04, 60.02, 62.57, 61.0, 61.0) | | | | |  |  |
| 16 | West Greenland | MarineRegions:iho | MarineRegions | iho | iho.29 | Davis Strait | include | 4250 |
| 17 | Massachusetts | MarineRegions:world_bay_gulf | MarineRegions | world_bay_gulf | world_bay_gulf.228 | Massachusetts Bay | include | 18882 |
| 17 | Massachusetts | MarineRegions:world_bay_gulf | MarineRegions | world_bay_gulf | world_bay_gulf.25 | Cape Cod Bay | include | 17487 |

1. Read, A. J. Age at sexual maturity and pregnancy rates of harbour porpoises *Phocoena phocoena* from the Bay of Fundy. *Can. J. Fish. Aquat. Sci.* **47**, 561–565 (1990).

2. Recchia, C. A. & Read, A. J. Stomach contents of harbour porpoises, *Phocoena phocoena (L.)*, from the Bay of Fundy. *Can. J. Zool.* **67**, (1989).

3. Halpern, B. S. *et al.* Recent pace of change in human impact on the world’s ocean. *Sci. Rep.* **9**, 1–8 (2019).

4. Gaskin, D. E., Frank, R. & Holdrinet, M. Polychlorinated biphenyls in harbor porpoises *Phocoena phocoena (L.)* from the Bay of Fundy, Canada and adjacent waters, with some information on chlordane and hexachlorobenzene levels. *Arch. Environ. Contam. Toxicol.* **12**, 211–219 (1983).

5. IWC. POP Contaminants in Cetacean Blubber - Trend explorer. *Sea Mammal Research Unit, University of St Andrews* http://www.smru.st-andrews.ac.uk/IWC_Contaminant_Explorer_POPs_V2/ (2019).

6. Murphy, S. *et al.* Spatio-temporal variability of harbour porpoise life history parameters in the North-east Atlantic. *Front. Mar. Sci.* **7**, (2020).

7. Hernández-Milián, G. Trophic role of small cetaceans and seals in Irish waters. (University of Cork, Ireland, 2014).

8. Kuiken, T. *et al.* PCBs, cause of death and body condition in harbour porpoises (<i>Phocoena phocoena<i/>) from British waters. *Aquat. Toxicol.* **28**, 13–28 (1994).

9. Law, R. J. *et al.* Chlorobiphenyls in the blubber of harbour porpoises (<i>Phocoena phocoena<i/>) from the UK: Levels and trends 1991-2005. *Mar. Pollut. Bull.* **60**, 470–473 (2010).

10. Sørensen, T. B. & Kinze, C. C. Reproduction and reproductive seasonality in Danish harbour porpoises, *Phocoena phocoena*. *Ophelia* **39**, 159–176 (1994).

11. Santos, M. B. *et al.* Harbor porpoise (*Phocoena phocoena*) feeding ecology in the eastern North Sea. *Int. Counc. Explor. Sea C. 15* (2005).

12. Granby, K. & Kinze, C. C. Organochlorines in Danish and West Greenland harbour porpoises. *Mar. Pollut. Bull.* **22**, 458–462 (1991).

13. Kleivane, L., Skaare, J. U., Bjorge, A., de Ruiter, E. & Reijders, P. J. H. Organochlorine pesticide residue and PCBs in harbour porpoises (*Phocoena phocoena*) incidentally caught in Scandinavian waters. *Environ. Pollut.* **89**, 137–146 (1995).

14. North Atlantic Marine Mammal Commission and the Norwegian Institute of Marine Research. Report of the Joint IMR/NAMMCO International workshop on the status of harbour porpoises in the North Atlantic. 236 (2019).

15. Weijs, L., van Elk, C., Das, K., Blust, R. & Covaci, A. Persistent organic pollutants and methoxylated PBDEs in harbour porpoises from the North Sea from 1990 until 2008: Young wildlife at risk? *Sci. Total Environ.* **409**, 228–237 (2010).

16. van den Heuvel-Greve, M. J. *et al.* Polluted porpoises: Generational transfer of organic contaminants in harbour porpoises from the southern North Sea. *Sci. Total Environ.* **796**, 148936 (2021).

17. van den Heuvel-Greve, M. J., IJsseldijk, L. L., Kwadijk, C. & Kotterman, M. *Contaminants in harbour porpoises beached along the Dutch coast*. (2018).

18. Murphy, S. *et al.* Assessing the effect of persistent organic pollutants on reproductive activity in common dolphins and harbour porpoises. *J. Northwest Atl. Fish. Sci.* **42**, 153–173 (2009).

19. Van Scheppingen, W. B., Verhoeven, A. J. I. M., Mulder, P., Addink, M. J. & Smeenk, C. Polychlorinated biphenyls, dibenzo-p-dioxins, and dibenzofurans in harbor porpoises (*Phocoena phocoena*) stranded on the Dutch coast between 1990 and 1993. *Arch. Environ. Contam. Toxicol.* **30**, 492–502 (1996).

20. Richardson, S. F. Growth and reproduction in the harbor porpoise, *Phocoena phocoena (L.)*, from Eastern Newfoundland. (Memorial University of Newfoundland, 1992).

21. Fontaine, P. M., Hammill, M. O., Barrette, C. & Kingsley, M. C. Summer diet of the harbor porpoise (*Phocoena phocoena*) in the estuary and the northern Gulf of St. Lawrence. *Can. J. Fish. Aquat. Sci.* **51**, 172–178 (1994).

22. Westgate, A. J., Muir, D. C. G., Gaskin, D. E. & Kingsley, M. C. S. Concentrations and accumulation patterns of organochlorine contaminants in the blubber of harbour porpoises, *Phocoena phocoena*, from the coast of Newfoundland, the Gulf of St Lawrence and the Bay of Fundy/Gulf of Maine. *Environ. Pollut.* **95**, 105–119 (1997).

23. Martin, A. R. The diet of harbor porpoises (*Phocoena phocoena*) in British waters. *Int. Whal. Comm.* **SC/47/SM48**, (1996).

24. Jepson, P. D. *et al.* PCB pollution continues to impact populations of orcas and other dolphins in European waters. *Sci. Rep.* **6**, 1–17 (2016).

25. Kesselring, T., Viquerat, S., Brehm, R. & Siebert, U. Correction: Coming of age: - Do female harbour porpoises (*Phocoena phocoena*) from the North Sea and Baltic Sea have sufficient time to reproduce in a human influenced environment? (PLoS One (2017) 12:10 (e0186951) DOI: 10.1371/journal.pone.0186951). *PLoS One* **13**, 1–14 (2018).

26. Gilles, A., Andreasen, H., Müller, S. & Siebert, U. *Nahrungsökologie von marinen Säugetieren und Seevögeln für das Management von NATURA 2000 Gebieten. Teil: Marine Säugetiere. Final report, submitted to the German Federal Agency for Nature Conservation (BfN)*. (2008).

27. Strandberg, B., Strandberg, L., Bergqvist, P. A., Falandysz, J. & Rappe, C. Concentrations and biomagnification of 17 chlordane compounds and other organochlorines in harbour porpoise (*Phocoena phocoena*) and herring from the southern Baltic Sea. *Chemosphere* **37**, 2513–2523 (1998).

28. Berggrena, P. *et al.* Patterns and levels of organochlorines (DDTs, PCBs, non-ortho PCBs and PCDD/Fs) in male harbour porpoises (*Phocoena phocoena*) from the Baltic Sea, the Kattegat-Skagerrak Seas and the West Coast of Norway. *Mar. Pollut. Bull.* **38**, 1070–1084 (1999).

29. Read, A. J. & Hohn, Aletta, A. Life in the fast lane: The life history of harbor porpoises from the Gulf of Maine. *Mar. Mammal Sci.* **11**, 423–440 (1995).

30. Gannon, D. P., Craddock, J. E. & Read, A. J. Autumn food habits of harbor porpoises, *Phocoena phocoena*, in the Gulf of Main. *Fish. Bull.* **96**, 428–437 (1998).

31. Tilbury, K. L., Stein, J. E., Meador, J. P., Krone, C. A. & Chan, S. L. Chemical contaminants in harbor porpoise (*Phocoena phocoena*) from the North Atlantic coast: Tissue concentrations and intra- and inter-organ distribution. *Chemosphere* **34**, 2159–2181 (1997).

32. Ólafsdóttir, D., Víkingsson, G. A., Halldórsson, S. D. & Sigurjónsson, J. Growth and reproduction in harbour porpoises (*Phocoena phocoena*) in Icelandic waters. *NAMMCO Sci. Publ.* **5**, 195 (2003).

33. Víkingsson, G. A., Ólafsdóttir, D. & Sigurjónsson, J. Geographical, and seasonal variation in the diet of harbour porpoises (*Phocoena phocoena*) in Icelandic coastal waters. *NAMMCO Sci. Publ.* **5**, 243 (2003).

34. Beineke, A. *et al.* Investigations of the potential influence of environmental contaminants on the thymus and spleen of harbor porpoises (*Phocoena phocoena*). *Environ. Sci. Technol.* **39**, 3933–3938 (2005).

35. Hedlund, H. Life history of the harbour porpoise (<i>Phocoena phocoena<i/>) in the Kattegat and Skaggerak Seas. (Stockholm, 2008).

36. Börjesson, P., Berggren, P. & Ganning, B. Diet of harbor porpoises in the Kattegat and Skagerrak seas: Accounting for individual variation and sample size. *Mar. Mammal Sci.* **19**, 38–058 (2003).

37. Polacheck, T., Wenzel, F. W. & Early, G. *What do stranding data say about harbor porpoises (<i>Phocoena phocoena<i/>)?* (1995).

38. Orphanides, C. D., Wenzel, F. W. & Collie, J. S. Diet of harbor porpoises (*Phocoena phocoena*) on the continental shelf off southern New England. *Fish. Bull.* **118**, 184–197 (2020).

39. Bjørge, A. Area Status Report of Norwegian waters north of 62N and western Russian waters. *Jt. IMR/NAMMCO Int. Work. STATUS Harb. PORPOISES NORTH Atl.* **Annex 6**, (2019).

40. Saint-André, C. The feeding ecology of harbor porpoises (*Phocoena phocoena*) in Norwegian coastal communities: a combined approach using stable isotope and stomach content analyses. (Master’s thesis, UiT Norges arktiske universitet, 2019).

41. Read, F. L. *et al.* Understanding harbour porpoise (Phocoena phocoena) and fisheries interactions in the north-west Iberian Peninsula. *Final Rep. to ASCOBANS* (2010).

42. Méndez-Fernandez, P. *et al.* An assessment of contaminant concentrations in toothed whale species of the NW Iberian Peninsula: Part I. Persistent organic pollutants. *Sci. Total Environ.* **484**, 196–205 (2014).

43. Norman, S. A. *et al.* Conception, fetal growth, and calving seasonality of harbor porpoise (*Phocoena phocoena*) in the Salish Sea waters of Washington, USA, and southern British Columbia, Canada. *Can. J. Zool.* **96**, 566–575 (2018).

44. Nichol, L. M. *et al.* Dietary overlap and niche partitioning of sympatric harbour porpoises and dall’s porpoises in the salish sea. *Prog. Oceanogr.* **115**, 202–210 (2013).

45. Calambokidis, J. & Barlow, J. A. Y. Chlorinated hydrocarbon concentrations and their use for describing population discreteness in harbor porpoises from Washington, Oregon, and California. *Mar. mammal strandings United States Proc. Second Mar. Mamm. Stranding Work.* **98**, 101–110 (1991).

46. Jarman, W. M. *et al.* Levels of organochlorine compounds, including PCDDS and PCDFS, in the blubber of cetaceans from the west coast of North America. *Mar. Pollut. Bull.* **32**, 426–436 (1996).

47. Learmonth, J. A. *et al.* Life history of harbor porpoises (*Phocoena phocoena*) in Scottish (UK) waters. *Mar. Mammal Sci.* **30**, 1427–1455 (2014).

48. Santos, M. B. *et al.* Variability in the diet of harbor porpoises (*Phocoena phocoena*) in Scottish waters 1992-2003. *Mar. Mammal Sci.* **20**, 1–27 (2004).

49. Wells, D. E., Campbell, L. A., Ross, H. M., Thompson, P. M. & Lockyer, C. H. Organochlorine residues in harbour porpoise and bottlenose dolphins stranded on the coast of Scotland, 1988-1991. *Sci. Total Environ.* **151**, 77–99 (1994).

50. Lockyer, C., Heide-Jørgensen, M. P., Jensen, J., Kinze, C. C. & Buus Sørensen, T. Age, length and reproductive parameters of harbour porpoises Phocoena phocoena (L.) from West Greenland. *ICES J. Mar. Sci.* **58**, 154–162 (2001).

51. Borrell, A. *et al.* Organochlorine residues in harbour porpoises from Southwest Greenland. *Environ. Pollut.* **128**, 381–391 (2004).

52. Lockyer, C. Harbour porpoises (*Phocoena phocoena*) in the North Atlantic: Biological parameters. *NAMMCO Sci. Publ.* **5**, 71 (2003).

53. Clausen, B. & Andersen, S. Evaluation of bycatch and health Status of the harbour porpoise (Phocoena phocoena) in Danish waters. *Danish Review of Game Biology* vol. 13 1–22 (1988).

54. DeMaster, D. P. Calculation of the average age of sexual maturity in marine mammals. *J. Fish. Res. Board Canada* **35**, 912–915 (1978).

55. Perrin, W. F., Holts, D. B. & Miller, R. B. Growth and reproduction of the eastern spinner dolphin, a geographical form of <i>Stenella longirostris<i/> in the eastern tropical Pacific. *Fish. Bull.* **75**, 725–750 (1977).

56. Hohn, Aletta, A. Variation in life history traits: the influence of introduced variation. (University of California, Los Angeles, 1989).

57. Bjørge, A. & Kaarstad, S. E. Age and body length at attainment of sexual maturityin harbour porpoise (*Phocoena phocoena*) in Norwegian and Swedish waters. *Sci. Comm. IWC* **SC/47/SM/7**, (1996).

58. Murphy, S. *et al.* Spatio-temporal variability of harbour porpoise life history parameters in English and Welsh waters. *Front. Mar. Sci.* (2020).

59. Frie, A. K., Stenson, G. B. & Haug, T. Long-term trends in reproductive and demographic parameters of female northwest atlantic hooded seals (<i>Cystophora cristata<i/>): Population responses to ecosystem change? *Can. J. Zool.* **90**, 376–392 (2012).

60. Abitia-Cardenas, L. A., Galvan-Magaña, F. & Rodriguez-Romero, J. Food habits and energy values of prey of striped marlin, *Tetrapturus audax*, off the coast of Mexico. *Fish. Bull.* **95**, 360–368 (1997).

61. Andersen, N. G. The effects of predator size, temperature, and prey characteristics on gastric evacuation in whiting. *J. Fish Biol.* **54**, 287–301 (1999).

62. Anthony, J. A., Roby, D. D. & Turco, K. R. Lipid content and energy density of forage fishes from the northern Gulf of Alaska. *J. Exp. Mar. Bio. Ecol.* **248**, 53–78 (2000).

63. Becker, B. H., Peery, M. Z. & Beissinger, S. R. Ocean climate and prey availability affect the trophic level and reproductive success of the marbled murrelet, an endangered seabird. *Mar. Ecol. Prog. Ser.* **329**, 267–279 (2007).

64. Boran, G. & Karaçam, H. Seasonal changes in proximate composition of some fish species from the black sea. *Turkish J. Fish. Aquat. Sci.* **11**, 01–05 (2011).

65. Carss, D. N. & Marquiss, M. The influence of a fish farm on grey heron Ardea cinerea breeding performance. *Aquat. predators their prey* 133–141 (1996).

66. Cumminns, K. W. & Wuycheck, J. C. Caloric equivalents for investigations in ecological energetics. *SIL Commun. 1953-1996* **18**, 1–158 (1971).

67. Eder, E. B. & Lewis, M. N. Proximate composition and energetic value of demersal and pelagic prey species from the SW Atlantic Ocean. *Mar. Ecol. Prog. Ser.* **291**, 43–52 (2005).

68. Elliott, K. H. & Gaston, A. J. Mass–length relationships and energy content of fishes and invertebrates delivered to nestling Thick-billed Murres. *Mar. Ornithol.* **36**, 25–34 (2008).

69. Friis, L. W., Jensen, Asger, H., Molzen, J. & Rebsdorf, M. Harbour seal diet in the central broads of Limfjorden, Denmark. *Flora og fauna* **100**, 57–62 (1994).

70. Härkönen, T. & Heide-Jørgensen, M. P. The harbour seal *Phoca vitulina* as a predator in the skagerrak. *Ophelia* **34**, 191–207 (1991).

71. Harris, M. P. & Hislop, J. R. G. The food of young Puffins *Fratercula arctica*. *J. Zool.* **185**, 213–236 (1978).

72. Harris, M. P., Newell, M., Daunt, F. & Speakman, J. R. Short communication aequoreus are poor food for seabirds. 413–415 (2008).

73. Lahaye, V. *et al.* Long-term dietary segregation of common dolphins. *Mar. Ecol. Prog. Ser.* **305**, 275–285 (2005).

74. Lawson, J. W., Hare, A., Noseworthy, E. & Friel, J. K. Assimilation efficiency of captive ringed seals (Phoca hispida) fed different diets. *Polar Biol.* **18**, 107–111 (1997).

75. Lawson, J. W., Magalhães, A. M. & Miller, E. H. Important prey species of marine vertebrate predators in the northwest Atlantic: Proximate composition and energy density. *Mar. Ecol. Prog. Ser.* **164**, 13–20 (1998).

76. Madigan, D. J. *et al.* Mercury stable isotopes reveal influence of foraging depth on mercury concentrations and growth in pacific bluefin tuna. *Environ. Sci. Technol.* **52**, 6256–6264 (2018).

77. Mårtensson, P. E., Nordøy, E. S. & Blix, A. S. Digestibility of crustaceans and capelin in harp seals (*Phoca groenlandica*). *Mar. Mammal Sci.* **10**, 325–331 (1994).

78. Martins, I., Pereira, J. C., Ramos, J. A. & Jørgensen, S. E. Modelling the effects of different quality prey fish species and of food supply reduction on growth performance of Roseate Tern chicks. *Ecol. Modell.* **177**, 95–106 (2004).

79. Meynier, L. *et al.* Intraspecific dietary variation in the short-beaked common dolphin *Delphinus delphis* in the Bay of Biscay: Importance of fat fish. *Mar. Ecol. Prog. Ser.* **354**, 277–287 (2008).

80. Montevecchi, W. A. & Piatt, J. Composition and energy contents of mature inshore spawning capelin (*Mallotus villosus*): Implications for seabird predators. *Comp. Biochem. Physiol. -- Part A Physiol.* **78**, 15–20 (1984).

81. Mr̊rtensson, P.-E., Gotaas, A. R. L., Norddy, E. S. & Blix, A. S. Seasonal Changes in Energy Density of Prey of Northeast Atlantic Seals and Whales. *Mar. Mammal Sci.* **12**, 635–640 (2006).

82. Murie, D. J. & Lavigne, D. M. Food consumption of wintering harp seals, *Phoca groenlandica*, in the St. Lawrence estuary, Canada. *Can. J. Zool.* **69**, 1289–1296 (1991).

83. Nelson, K. & Kruuk, H. The prey of otters: Calorific content of eels (*Anguilla anguilla*) and other fish, frogs (*Rana temporaria*) and toads (*Bufo bufo*). *IUCN Otter Spec. Gr. Bull.* **14**, 75–80 (1997).

84. Nolet, B. A. & Kruuk, H. Grooming and resting of otters *Lutra lutra* in a marine habitat. *J. Zool.* **218**, 433–440 (1989).

85. Norman, J. R. A history of fishes. *Ernest Benn Ltd. London* (1963).

86. Pedersen, J. & Hislop, J. R. G. Seasonal variations in the energy density of fishes in the North Sea. *J. Fish Biol.* **59**, 380–389 (2001).

87. Perez, M. A. *Calorimetry measurements of energy value of some Alaskan fishes and squids*. *NOAA Technical Memorandum* vol. NMFS-AFSC- (1994).

88. Rosen, D. A. S. & Trites, A. W. Digestive efficiency and dry-matter digestibility in Steller sea lions fed herring, pollock, squid, and salmon. *Can. J. Zool.* **78**, 234–239 (2000).

89. Sánchez, S., Palomera, I., Albo-Puigserver, M. & Bernal, M. Energy density and lipid content of sardine (Sardina pilchardus) and anchovy (Engraulis encrasicolus) in the Catalan Sea, Northwestern Mediterranean Sea. in *40th CIESM Congress: The largest Forum on Mediterranean and Black Sea Research* vol. 40 530 (2013).

90. Schreckenbach, Knösche & Ebert. Nutrient and energy content of freshwater fishes. *J. Appl. Ichthyol.* **17**, 142–144 (2008).

91. Spitz, J. & Jouma’a, J. Variability in energy density of forage fishes from the Bay of Biscay (north-east Atlantic Ocean): Reliability of functional grouping based on prey quality. *J. Fish Biol.* **82**, 2147–2152 (2013).

92. Spitz, J., Mourocq, E., Schoen, V. & Ridoux, V. Proximate composition and energy content of forage species from the Bay of Biscay: High- or low-quality food? *ICES J. Mar. Sci.* **67**, 909–915 (2010).

93. Steimle, F. W. & Terranova, R. J. Energy contents of northwest Atlantic continental slope organisms. *Deep Sea Res. Part A, Oceanogr. Res. Pap.* **35**, 415–423 (1988).

94. Steimle, F. W. J. & Russell, J. Energy equivalents of marine organisms from the continental shelf of the temperate Northwest Atlantic. *J. Northwest Atl. Fish. Sci.* **6**, 117–124 (1980).

95. Temming, A. & Herrmann, J. P. Gastric evacuation in cod. Prey-specific evacuation rates for use in North Sea, Baltic Sea and Barents Sea multi-species models. *Fish. Res.* **63**, 21–41 (2003).

96. Van Pelt, T. I., Piatt, J. F., Lance, B. K. & Roby, D. D. Proximate composition and energy density of some North Pacific forage fishes. *Comp. Biochem. Physiol. - A Physiol.* **118**, 1393–1398 (1997).

97. Whyte, J. N. C., Beamish, R. J., Ginther, N. G. & Neville, C.-E. Nutritional condition of the Pacific Lamprey (*Lampetra tridentata*) deprived of food for periods of up to two years. *Can. J. Fish. Aquat. Sci.* **50**, 591–599 (1993).

98. Frazier, M. Recent pace of change in human impact on the world’s ocean: Cumulative impacts. *Knowledge Network for Biocomplexity* https://knb.ecoinformatics.org/view/doi:10.5063/F12B8WBS (2019) doi:doi:10.5063/F12B8WBS.
